# A competitive precision CRISPR method to identify the fitness effects of transcription factor binding sites

**DOI:** 10.1038/s41587-022-01444-6

**Published:** 2022-09-26

**Authors:** Päivi Pihlajamaa, Otto Kauko, Biswajyoti Sahu, Teemu Kivioja, Jussi Taipale

**Affiliations:** 1 Applied Tumor Genomics Research Program, Faculty of Medicine, University of Helsinki, Helsinki, Finland; 2 Department of Biochemistry, University of Cambridge, Cambridge, UK; 3 Turku Bioscience Centre, University of Turku and Åbo Akademi University, Turku, Finland; 4 Medicum, Faculty of Medicine, University of Helsinki, Helsinki, Finland; 5 Department of Medical Biochemistry and Biophysics, Karolinska Institute, Stockholm, Sweden

**Keywords:** Transcription, Genetic engineering, Genetics

## Abstract

Here we describe a competitive genome editing method that measures the effect of mutations on molecular functions, based on precision CRISPR editing using template libraries with either the original or altered sequence, and a sequence tag, enabling direct comparison between original and mutated cells. Using the example of the MYC oncogene, we identify important transcriptional targets and show that E-box mutations at MYC target gene promoters reduce cellular fitness.

## Abstract (web summary)

A competitive CRISPR method discovers targets and phenotypic effects of the MYC oncogene.

## Main

The current genome editing tools, such as CRISPR–Cas9, have proven to be robust and efficient tools for many sequence manipulations. They have been extensively used for mutating specific genomic loci in single-gene studies^1^ as well as genome-wide screens^2–4^. However, resolution of the CRISPR–Cas9 editing is limited by the suitable protospacer adjacent motif (PAM) sequences found in close proximity of the region of interest. Homology-directed recombination (HDR)-mediated precision editing can be used to introduce genetic alterations exactly at the intended loci, but this method suffers from strong DNA damage response, low efficiency and incompatibility with pooled CRISPR screening approaches. Because of the low efficiency of precision genome editing, pooled screens commonly use lentiviral introduction of libraries of guide RNAs to cell lines that express either Cas9 nuclease alone that generates a series of insertion and deletion alleles or nuclease-dead Cas9 fused to transcriptional repressor (CRISPRi) or activator (CRISPRa) domains^5–7^. These methods do not have single-base or single-allele resolution, and their precision is limited because they use an indirect measure, inferring the perturbation from the presence of a guide sequence integrated into the cells at a (pseudo)random genomic position.

Furthermore, interpreting the functional consequence of targeted Cas9-induced mutations is confounded by the DNA damage introduced by Cas9 and the off-target effects of the Cas9 nuclease^8^. In particular, double-strand breaks (DSBs) at on-target or off-target loci cause DNA damage and genomic instability, resulting in paused cell cycle or apoptosis^9–11^. These problems are particularly acute in analysis of small intergenic features, such as transcription factor (TF) binding sites. This is because non-coding sequence is commonly repetitive, and single guide RNAs (sgRNAs) targeting small binding motifs cannot be selected from a large number of possible sequences predicted to have the same effect. Here we describe a competitive precision genome editing (CGE) approach using CRISPR–Cas9 genome editing at precise loci to accurately analyze the effect of mutations on cellular properties and molecular functions, such as fitness, TF binding and mRNA expression. The experimental design in the CGE approach mitigates the confounding factors associated with CRISPR experiments, such as the hampering effect of double-strand DNA break itself on cell proliferation, enabling dissection of the effect of individual sequence features on cellular fitness. Here, we use the CGE method for dissecting the transcriptional network downstream of the master regulatory oncogene MYC.

MYC is a basic helix-loop-helix (bHLH) TF that forms a heterodimer with another bHLH protein, MAX, and regulates a large set of target genes by binding to regulatory elements containing E-box (CACGTG) motifs^12–14^. MYC is indispensable for embryonic development^15^, but, in normal cells, its expression is tightly controlled. The importance of tight regulation of MYC activity is highlighted by the fact that it is one of the most frequently deregulated oncogenes across multiple human cancer types^16^. MYC regulates major pathways promoting cell growth and proliferation, such as ribosome biogenesis and nucleotide biosynthesis^17^. However, owing to the large number of MYC targets, identifying direct transcriptional targets of MYC has been challenging. It has been proposed that MYC, instead of being a regulator of a particular transcriptional programs, is a universal amplifier of gene expression that increases transcriptional output at all active promoters^18,19^. Conversely, it has been shown that MYC can selectively regulate specific sets of genes, including those involved in metabolism and assembly of the ribosome^20–22^. Nevertheless, despite its well-known phenotypic effects on cellular growth and proliferation, the precise MYC target genes accounting for its oncogenic activity are still elusive. We reasoned that the most effective way to dissect the gene regulatory network downstream of MYC would be to individually assess the role of each target gene by mutating the MYC binding sites at its regulatory regions, which we have done here using the CGE method.

**Fig. 1:**
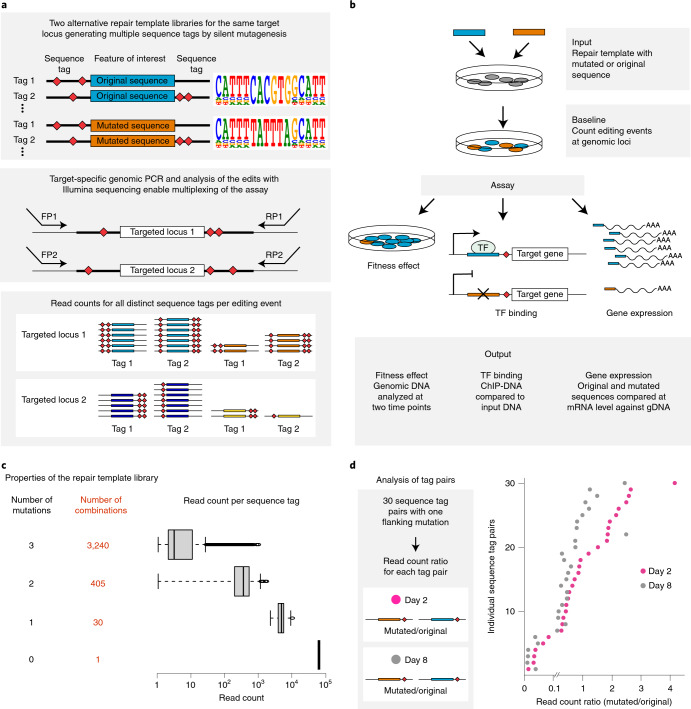
Strategy of the CGE method to lineage-trace cells with distinct genome editing events using sequence tags with silent or near-silent mutations. **a**, The CGE method uses a library of HDR templates with two experimental variants: original genomic sequence (blue) and desired mutation (orange). In addition, the HDR templates harbor sequence tags that can be identified by Illumina sequencing of the targeted locus, enabling lineage tracing of the edited clones and creating a large number of internal replicates in each experiment. The sequence tags are generated by mutating nucleotides flanking the region of interest with the probability of 24%, a strategy that typically introduces 2–3 mutated nucleotides (indicated with red diamonds; Extended Data Fig. 1), leaving most of the flanking sequence intact, as demonstrated by the position weight matrices. **b**, Experimental strategy using a mixture of HDR template libraries harboring the original and mutated sequences for the same target. The abundance of each HDR template in the cell population is analyzed from the sequence tags after different assays and compared to respective baseline: cellular fitness (gDNA at day 8/day 2), TF binding (chromatin-immunoprecipitated DNA/input DNA) and mRNA expression (mRNA abundance/respective gDNA). **c**, The number of possible sequence variations with zero (*n* = 1), one (*n* = 30), two (*n* = 405) and three (*n* = 3,240) flanking mutations when the sequence tags are created by mutating ten nucleotides with the probability of 24% and their abundance in the HDR template library analyzed from read counts in ChIP input sample of the edited SHMT2 E-box locus. The box plots indicate the median read count with upper and lower quartiles, and the whiskers extend to 1.5 times the interquartile range. The number of sequence tags recovered in each experiment is shown in Supplementary Table 3. **d**, The effect of E-box mutation at the *RPL23* gene promoter on fitness of HAP1 cells shown by read count ratios for mutated/original sequences for each cell lineage pair harboring identical sequence tags with one flanking mutation (see also Extended Data Figs. 1b and 2b). Of note, the sequence tags with two flanking mutations are used in Fig. 2 for more robust analysis (Methods).

The CGE method uses CRISPR–Cas9 technology combined with a library of HDR templates with sequence tags enabling lineage tracing of the targeted cell populations. The HDR templates harbor two types of mutations: experimental variants targeting a genomic feature of interest and silent or near-silent mutations that introduce variable sequence tags (Fig. 1a). One of the key design features of the CGE method is the use of at least two experimental variants. One of them (control) reconstitutes the wild-type sequence of the region of interest by harboring the original genomic sequence, whereas the other replaces it with desired mutated sequence, such as non-functional TF binding site (Fig. 1a). In addition to the experimental variants, each individual HDR template molecule has variable sequence tag(s) flanking the sequence of interest serving as a genetic barcode that can be detected from the Illumina sequencing reads of the targeted locus (Fig. 1a). Inclusion of a large set of different sequence tags generates a large number of internal replicate lineages and lineage pools within each assay. As most cells remain unedited, the lineages are expected to grow largely independently of each other, increasing the statistical power of the method. Inclusion of the tags also allows precise counting of the editing events and enables exclusion of the possibility that the tags themselves, and not the intended mutations, cause the observed phenotype. Pairwise analysis of the cell lineages harboring the same sequence tags, in turn, enables direct measurement of the effect of the targeted mutation.

In the CGE experiment, DNA samples from cells edited with either mutant or control sequence are collected at two or more timepoints (early and late), and the cell lineages with particular editing event can be followed before and after subjecting the cells to selection pressure, such as competitive growth in culture, after which cellular fitness can be analyzed (Fig. 1b). In addition, the CGE method can be used for measuring the effect of defined mutations on TF binding to the target locus and on the expression levels of mRNA by comparing the chromatin-immunoprecipitated DNA to input DNA or mRNA levels against respective genomic DNA (gDNA). Because the sequence tags are present in both repair templates, this experimental design allows precise comparison of the mutated versus control sequence by excluding the non-edited wild-type sequences from the analysis. Sequencing reads will then be assigned to the distinct editing events based on their sequence tags, and the ratio between mutated and control sequences for each tag are determined at each experimental condition (such as both timepoints), resulting in dozens of internal replicate measurements for each editing event within a single assay (Fig. 1a). Thus, statistical power to detect differences between the conditions is very high. The experiment is a single-well assay in which the repair templates harboring both experimental variants (mutant and control) are transfected to cells within one culture well, and the genomic perturbation is compared directly to control in the same cell population. This eliminates the experimental bias and variation originating from transfection/transduction and Cas9-introduced DSBs and variation caused by differences in culture and experimental conditions between wells. Thus, the CGE method is a sensitive assay with lower risk for systematic errors and fewer confounding variables compared to replicate experiments performed in separate wells.

**Extended Data Fig. 1:**
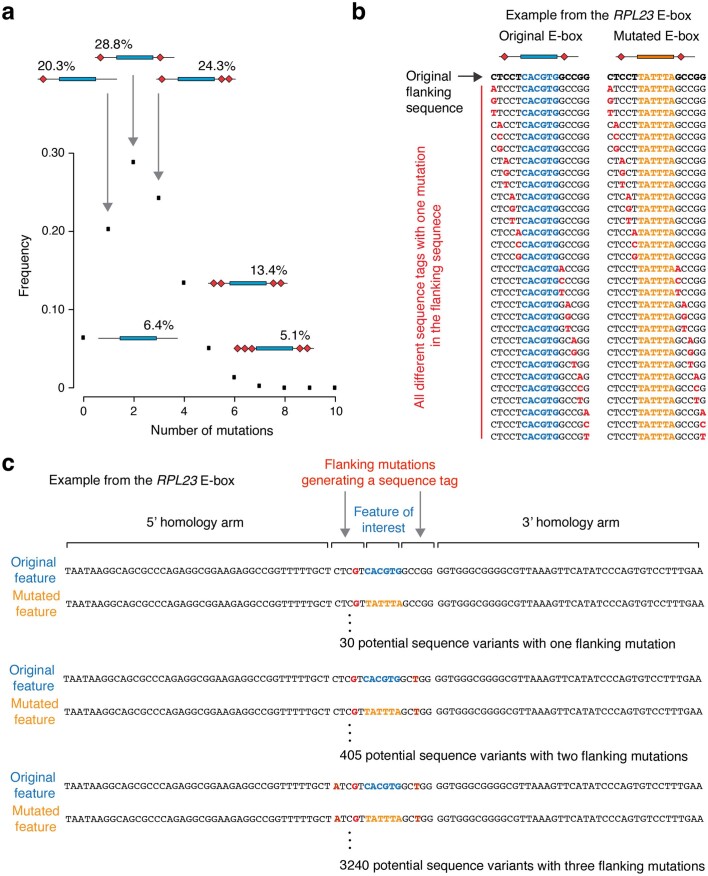
Generating sequence tags with silent mutations for non-coding genomic regions. **a**, Frequency of the flanking mutations in the HDR templates. For creating sequence tags with silent or near-silent mutations, each of the ten positions flanking the MYC binding motif (E-box) was mutated with probability of 24% (each of the three non-consensus nucleotides introduced with a probability of 8%). This results in a library of sequences with most of the flanking sequence intact; oligoes synthesized using this approach typically harbor one, two, or three flanking mutations with probability of 20.3, 28.8 and 24.3%, respectively. **b**, All variations for sequence tags harboring one mutation (indicated in red) within ten nucleotides flanking the original E-box sequence (CACGTG) or mutated sequence (TATTTA) in HDR oligoes targeting the MYC binding motif at the *RPL23* promoter. **c**, Examples of full length HDR oligos targeting the *RPL23* promoter. The constant regions serving as homology arms and the two types of mutations are shown: (1) the experimental variants targeting the sequence of interest (E-box) in the middle of the oligo with either original E-box sequence (blue) or mutated non-functional sequence (orange); (2) flanking mutations that generate variable sequence tags (mutated nucleotide indicated in red, consensus sequence in black). Examples of sequence tags with one, two, and three flanking mutations are shown; the total number of potential tags with these mutations is also indicated.

**Extended Data Fig. 2:**
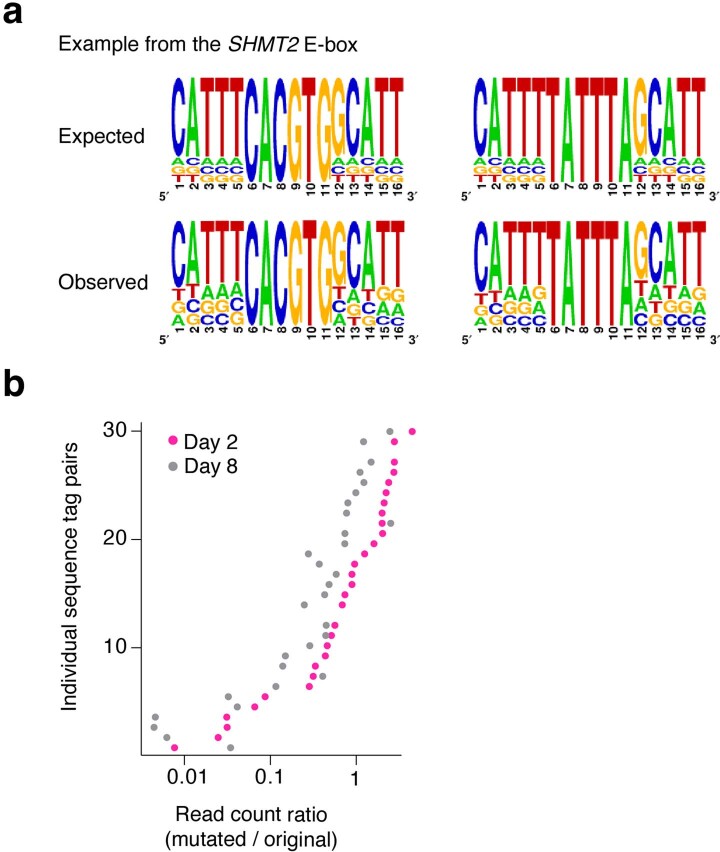
Sequence tags enable lineage-tracing of edited cells. **a**, Expected and observed sequence tags targeting the E-box at the *SHMT2* promoter. Sequence logos for expected distribution of flanking mutations were generated on the basis of the mutation strategy used for generating the library of HDR templates: each non-consensus base is represented with a probability of 8%. The observed sequence logos were generated from all sequence tags observed in the ChIP input sample for targeted SHMT2 E-box locus. The sequence tags within the HDR templates with original E-box sequence are shown on the left (from 8442 recovered tags), and with the mutated TATTTA sequence on the right (from 9128 recovered tags). ChIP input is collected 48 h after transfection and thus represents the baseline of transfected HDR templates in a single experiment. Note that the consensus bases are taller in the logo representing the expected sequences compared to the observed ones, since expected sequences also include the variants without any flanking mutations (that are inevitably generated in this mutation strategy), whereas the observed logos are generated for the sequence tags with at least one mutation. **b**, Precision editing results for the effect of E-box mutation at the *RPL23* gene promoter on fitness of HAP1 cells separately for each cell lineage pair with a sequence tag having exactly one flanking mutation (x-axis on logarithmic scale, see also Fig. 1d). Read count ratios for mutated vs. original sequence are shown at two time points, day 8 (grey) and day 2 (pink).

To preserve potentially functional flanks of the sequence of interest, it is important that the sequence tags are introduced using silent or near-silent mutations. For coding regions, this can be accomplished by introducing synonymous mutations of codons and avoiding splice junctions. Because less is known about functional elements within non-coding regions, we decided to use a diverse library that largely conserves the wild-type sequence, introducing only one or few point mutations per cell within the five nucleotides flanking the sequence of interest on both sides, a region wider than a typical TF binding site (~10 base pairs (bp)). In our case, each of the ten positions within the flanking sequence was mutated with probability of 24%, thus keeping most of the positions intact (Fig. 1a) but introducing typically (in ~53% of the sequences) 2–3 mutations per repair oligo (Extended Data Fig. 1a). This mutation strategy generates 30 distinct sequence tags whose sequence differs from the native sequence by exactly one nucleotide (Extended Data Fig. 1b), 405 distinct sequences with 2-nucleotide (nt) difference to the native sequence and 3,240 distinct sequences with three mutations (Fig. 1c and Extended Data Fig. 1c). In the oligo synthesis for HDR templates, the probability for any individual sequence tag with one mutation is higher than for tag with two or three mutations, which is reflected in the data with single-mutation tags having higher read counts than double and triple mutants (Fig. 1c), consistent with the fact that single-mutation sequence tags are present in the original mixture of synthesized oligos in more copies than double and triple mutants. Control experiments also indicated that the overall base distribution of the flanking mutations at day 2 was fairly uniform (Extended Data Fig. 2a). After assigning the read counts for each cell lineage with a unique sequence tag and distinct experimental mutation (mutated or native sequence of interest) at the two experimental timepoints, a pairwise analysis for the cell lineages harboring identical sequence tags can be performed by calculating the ratio of mutated-to-native sequences for each sequence tag pair. This mitigates the potential effect of the flanking mutation on the measured phenotype and enables robust and accurate measurement for the effect of the mutation on cellular fitness for each cell lineage separately (Fig. 1d and Extended Data Fig. 2b).

To validate our CGE approach in functional studies, we first introduced mutations to the coding regions of genes. To this end, we mutated previously described phosphorylation sites of the *CDK1* (cyclin-dependent kinase 1) and the *GRB2* (growth factor receptor-binding protein 2) genes. In coding regions, sequence tags were generated by randomizing the degenerate positions of the adjacent codons in the repair template. Phosphorylation sites were abolished by alanine (A) or phenylalanine (F) substitutions of the phosphorylated serine (S), threonine (T) or tyrosine (Y) residues. To mimic phosphorylation, the same amino acids were also mutated to the acidic residues glutamate (E) or aspartate (D), which, in many proteins, can lead to the same effect as phosphorylation of the serine, threonine or tyrosine residues^23^. In the CGE method, the cell lineages that carry mutations that impair cell proliferation should be underrepresented in the cell population after 1 week of culture compared to cells edited with the original sequence with the same sequence tags. This can be analyzed from gDNA collected at the beginning and at the end of the experiment (Fig. 1b).

**Extended Data Fig. 3:**
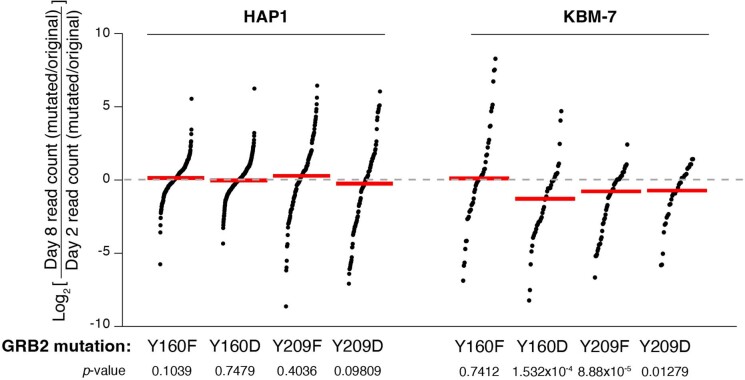
The effects of mutating phosphorylation sites in the *GRB2* gene on cellular fitness of HAP1 and KBM-7 cells. Precision editing results are shown for all cell lineages harboring sequence tags with read count >20 on day 2. Log_2_ values for read count ratios (day 8/day 2) are shown for each sequence tag pair after calculating the ratio of read counts for mutated vs. original sequence at each time point. Black dots represent independent cell lineages (internal replicates); red lines mark the median values, and *p*-values from two-sided Wilcoxon signed rank test are shown for each experiment with no multiple comparison adjustments (see Supplementary Table 3 for details of statistical parameters).

The experiments for measuring the effect of phosphorylation sites in the GRB2 protein were carried out in haploid HAP1 and near-haploid chronic myelogenous leukemia KBM-7 cell lines. HAP1 cells are a derivative of KBM-7 that grow adherently, no longer express hematopoietic markers and, in early passage cultures, are haploid for all chromosomes. Haploid and near-haploid cells are particularly useful for mutational screens because only one editing event is sufficient for a full knockout. Previous mutagenesis screen by Blomen et al.^24^ suggests that the adaptor protein GRB2 that links tyrosine kinase signaling to the RAS-mitogen-activated protein kinase (MAPK) pathway is essential for both KBM-7 and HAP1 cells, but all other components of the BCR/ABL-RAS/MAPK pathway are only essential for KBM-7 but not for HAP1 cells. GRB2 is phosphorylated at Y160 and Y209, with phosphorylated Y160 activating and Y209 inhibiting downstream MAPK signaling^25,26^. Mutation Y160F to prevent activation of MAPK had no effect in either cell type, whereas the mutations Y160D and Y209F that are predicted to increase MAPK activity decreased proliferation of KBM-7 but not HAP1 cells (Extended Data Fig. 3), consistent with the more important role of RAS/MAPK signaling in KBM-7 compared to HAP1 cells. The decreased fitness observed for KBM-7 cells upon MAPK activation might result, for example, from MAPK-induced senescence^27,28^. These results indicate that the CGE method can be used to separate essentiality of a gene from essentiality of individual amino acid residues and to identify functionally important phosphorylation events in cells.

**Fig. 2:**
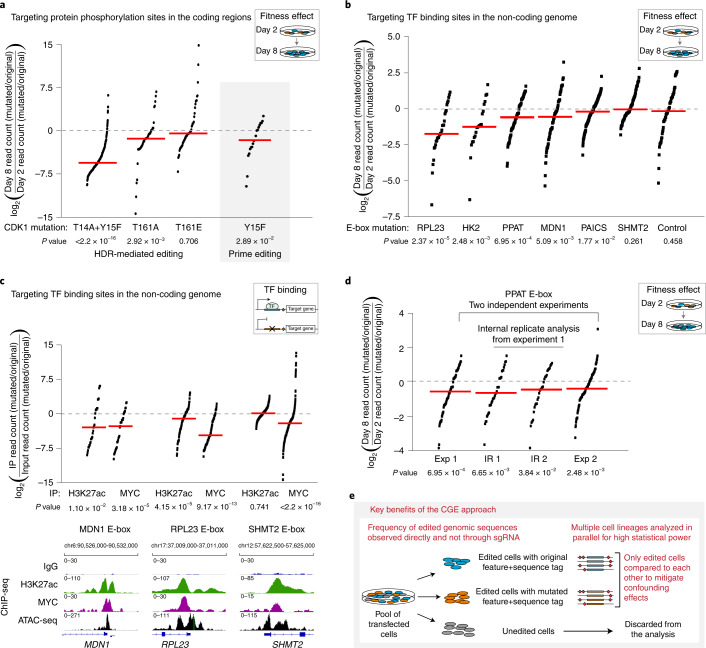
The effect of mutating TF binding sites and protein phosphorylation sites on cellular fitness determined by lineage tracing of editing events. **a**, The effect of mutating protein phosphorylation sites of CDK1 on fitness of HAP1 cells. log_2_(day 8/day 2) is shown for each sequence tag pair with read count >5 on day 2 after calculating the ratio of read counts for mutated/original sequences at both timepoints. The CGE method was used for measuring the effect of Y15F mutation on the fitness also after introducing this mutation to HAP1 cells using prime editing^33^. In **a**–**d**, dots represent individual cell lineages harboring a unique barcode—that is, internal replicates for which median (red line) and *P* value are calculated (two-sided Wilcoxon signed-rank test separately for each experiment, no multiple comparison adjustments; see Supplementary Table 3 for statistical details and Supplementary Table 4 for sequencing depth and editing efficiency). **b**, The effect of mutating MYC binding motifs (E-box) at promoters of MYC target genes on fitness of HAP1 cells (see also Extended Data Fig. 4)—synonymous mutation in the *MYC* coding region as a negative control. log_2_(day 8/day 2) is shown for each sequence tag pair with two flanking mutations and read count >50 on day 2 after calculating the ratio of read counts for mutated/original sequences at both timepoints (see also Supplementary Table 5). **c**, The effect of E-box mutation on MYC occupancy and H3K27ac at promoters of MYC target genes. log_2_(IP sample/input) is shown for each sequence tag pair with two flanking mutations and read count >100 in the input after calculating the ratio of read counts for mutated/original sequences. Genome browser snapshots with ChIP-seq and ATAC-seq tracks demonstrate robust MYC binding to the targeted sites in wild-type HAP1 cells. **d**, Reproducibility of the CGE method shown for the E-box at the *PPAT* promoter from two independent experiments (Exp 1 and Exp 2) and from two internal replicate groups (IR1 and IR2) (Methods). **e**, The key advantages of the CGE method are high statistical power due to internal replicates and mitigation of the confounding effects characteristic of CRISPR–Cas9-based methods by excluding the unedited cells.

To further validate our CGE method, we evaluated the fitness effect of CDK1 regulatory phosphorylation site mutations in human HAP1 cells. CDK1 activation and onset of mitosis requires phosphorylation of T161 in the activation segment and dephosphorylation of T14 and Y15 (ref. ^29^). The non-phosphorylatable double-mutant T14A/Y15F cells were almost completely lost after 1 week of precision editing (Fig. 2a). These findings are consistent with earlier work reporting that the T14A/Y15F double mutant can be activated prematurely during the cell cycle^30^, and overexpression of this mutant in cells results in cell death due to mitotic catastrophe^31^. The effect of the phosphorylation site mutation in the CDK1-activating segment, T161A, was less prominent. Loss of phosphorylation resulted in markedly decreased cell proliferation, whereas T161E phosphomimetic mutation allowed cells to proliferate normally (Fig. 2a). This is consistent with the lack of requirement of regulation of the CDK-activating kinase in human cells^32^. We also tested the recently reported prime editing method^33^ for mutating a phosphorylation site and for introducing the sequence tag within the *CDK1* coding region. Using this approach, we observed reduced fitness of HAP1 cells as a result of Y15F mutation (Fig. 2a), demonstrating that prime editing can also be used for generating the targeted mutations and sequence tags for our precision genome editing method.

**Extended Data Fig. 4:**
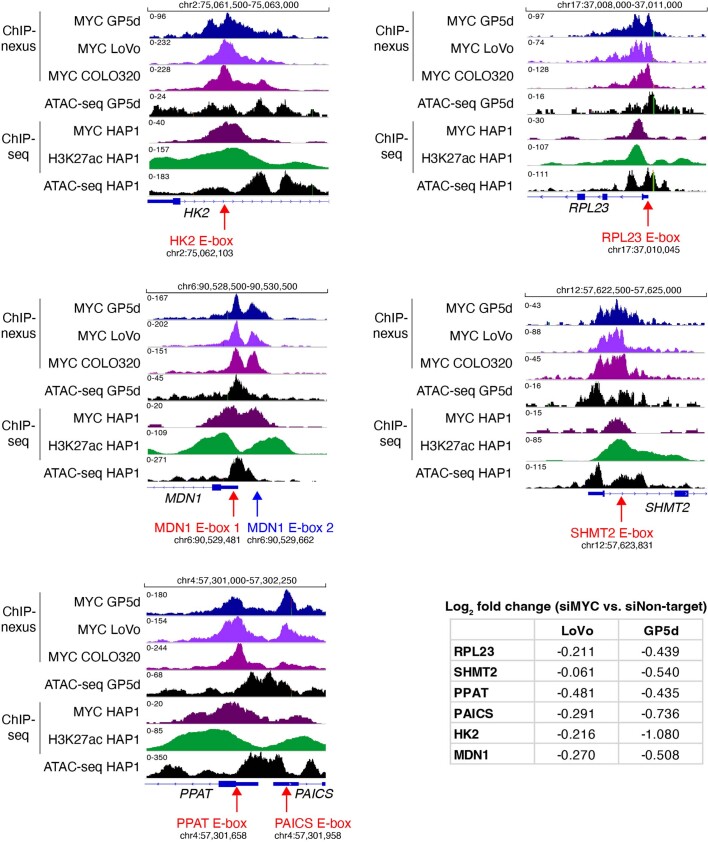
MYC target genes and the location of targeted E-boxes. Genome browser snapshots showing MYC binding at promoters of MYC target genes in three colon cancer cell lines GP5d, LoVo, and COLO320 and ATAC-seq signal for open chromatin in GP5d cells (traces from BAM coverage files are shown for ChIP-nexus tracks from ref. ^34^ and for GP5d ATAC-seq from ref. ^35^). The high spatial resolution of the ChIP-nexus peaks was utilized for selecting the MYC binding sites for the CGE experiments; the locations of E-boxes targeted in Fig. 2b are marked with red arrows and the additional MDN1 E-box targeted in Extended Data Fig. 5 is marked with a blue arrow (genome coordinates indicate the position of the first cytosine in the E-box sequence CACGTG in the forward strand; hg19). In addition, ChIP-seq tracks for MYC and H3K27ac as well as BAM coverage track for ATAC-seq from HAP1 cells is shown. Table shows differential gene expression for the selected MYC target genes as log_2_(fold change) values upon MYC silencing (siMYC) in LoVo and GP5d cells (RNA-seq data from ref. ^34^).

After demonstrating the power of the precision editing approach in studying the functional consequence of individual protein phosphorylation sites, we used it for studying the gene regulatory elements within the non-coding genome. Specifically, a 6-nt MYC binding motif (E-box) was mutated at the promoters of MYC target genes to study their effect on cell proliferation and fitness. If a particular E-box is essential for cell growth, the alleles containing tags and the wild-type sequence should be enriched in the cell population compared to the E-box deleted alleles after 1 week of culture (Fig. 1b,d). Although a large number of genes have been reported as MYC target genes^17^, the functional consequence for cell proliferation resulting from MYC binding to a promoter of a particular gene has not been previously shown. For the purpose of this study, putative MYC target genes were selected for editing on the basis of functional genomics studies in human colon cancer cell lines and previously published datasets in the HAP1 haploid cell line using the following criteria: (1) the gene should preferably contain only one E-box within the chromatin immunoprecipitation (ChIP)-nexus peak^34^ (Extended Data Fig. 4); (2) the gene should display robust MYC binding at its promoter within open chromatin on the basis of signal from assay for transposase-accessible chromatin with sequencing (ATAC-seq) and clear change in expression upon MYC silencing in colon cancer cells^34,35^ (Extended Data Fig. 4); and (3) the gene must be essential in HAP1 cells, reported by both publications^24,36^. Gene essentiality was used as a selection criterion because it is likely that fitness effects can be found for regulatory or epigenetic elements associated with essential genes. It should be noted, however, that individual binding motif mutations are likely to cause less severe phenotypes than loss of entire genes, as single binding motifs may contribute only partially to gene expression or not be required for expression at all. Thus, CGE targeting of binding motifs does not address the essentiality of the target genes per se but can be used for identifying critical regulatory or epigenetic features controlling the function of these genes.

**Extended Data Fig. 5:**
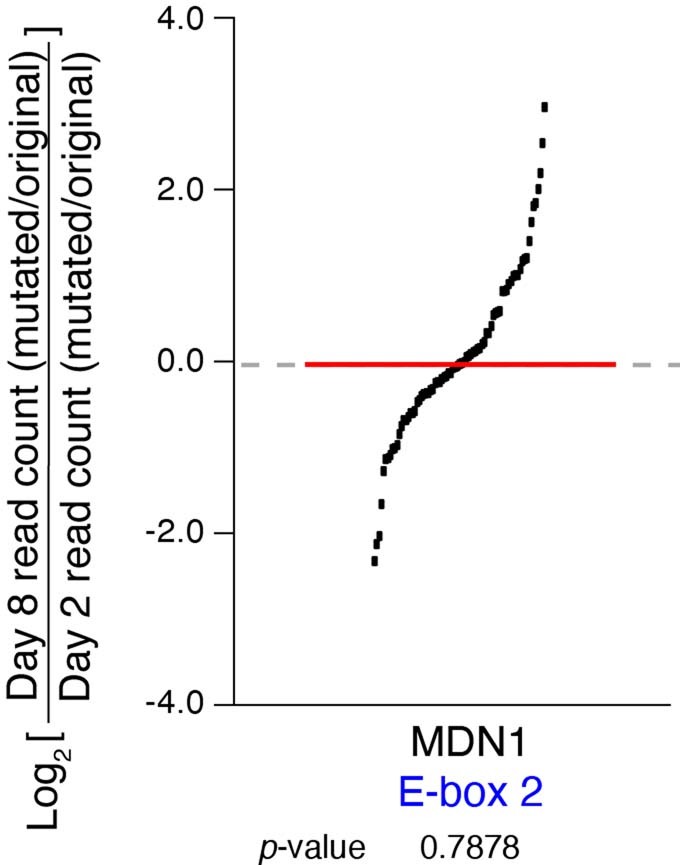
The effect of mutating the second E-box at the *MDN1* promoter on cellular fitness of HAP1 cells. Log_2_ values for read count ratios (day 8/day 2) are shown for each sequence tag pair after calculating the ratio of read counts for mutated vs. original sequence at each time point. Red line indicates the median, and *p*-value from two-sided Wilcoxon signed rank test is shown, no multiple comparison adjustments (see Extended Data Fig. 4 for location of the targeted E-box; Supplementary Table 3 for details of statistical parameters). Note that mutating the E-box closer to the TSS (E-box 1; TSS + 32) affects cellular fitness (see Fig. 2b), whereas targeting the one farther away (E-box 2; TSS -151) does not, as shown here.

The CGE experiments for testing the effect of E-box mutations were carried out in HAP1 cells using the original E-box sequence and a non-functional TATTTA sequence as the experimental variants and the flanking near-silent mutations as the sequence tags (Extended Data Fig. 1). For the different E-box targets, 7–42% of the sequencing reads matched to the mutation patterns expected from the HDR-mediated editing (Supplementary Table 4). The cell lineages harboring either the original or mutated sequence with exactly two flanking mutations were analyzed at day 2 and day 8 (Methods). Targeted mutation of the E-box sequence to a non-functional TATTTA at the promoters of four MYC target genes—*RPL23* (ribosomal protein L23), *HK2* (hexokinase 2), *PPAT* (phosphoribosyl pyrophosphate amidotransferase) and *MDN1* (midasin AAA ATPase 1)—resulted in reduced cell growth as measured from the read counts for the sequence tags with two mutations at day 8 as compared to day 2 (Fig. 2b). However, there were E-boxes at promoters of MYC target genes that can be mutated to non-functional sequence without affecting cell proliferation, such as *SHMT2* (serine hydroxymethyltransferase 2) and *PAICS* (phosphoribosylaminoimidazole carboxylase and phosphoribosylaminoimidazolesuccinocarboxamide synthase) (Fig. 2b), demonstrating the strength of this approach in dissecting the contribution of each individual TF binding site to cell proliferation. Furthermore, the CGE method can robustly measure the effect of each E-box on cellular fitness also for genes that harbor several of them within their regulatory region, as demonstrated for the *MDN1* gene. Out of the two E-boxes within the *MDN1* promoter, mutation of the E-box closer to the transcription start site (TSS) (TSS +32) had an effect on cell proliferation (Fig. 2b), whereas the mutation of the E-box farther away (TSS −151) had no effect (Extended Data Fig. 5), despite MYC binding detected at both of these sites in HAP1 cells as well as using ChIP-nexus in colon cancer cells^34^ (Extended Data Fig. 4).

**Extended Data Fig. 6:**
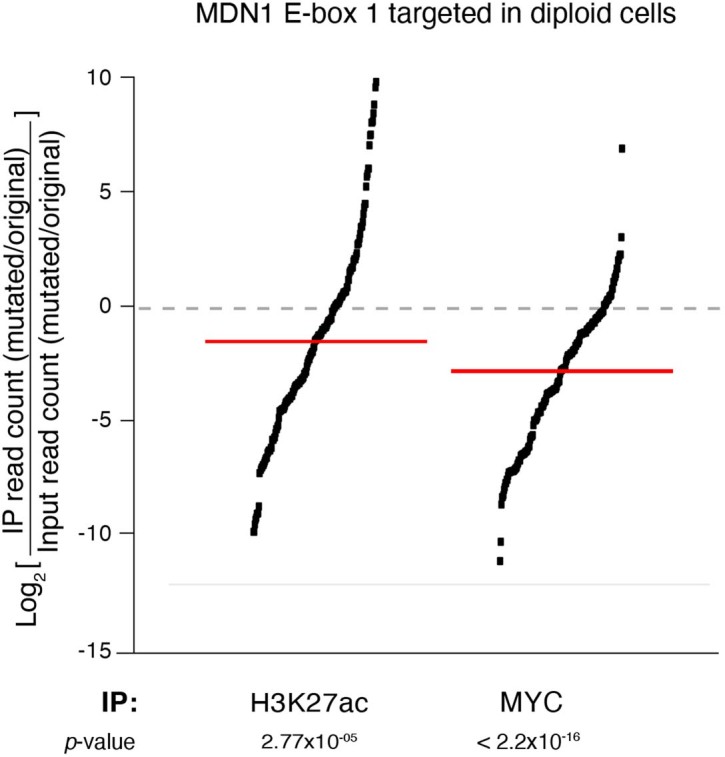
The effect of E-box mutation on MYC occupancy and acetylation of H3K27 at the *MDN1* promoter in HCT116 cells. ChIP using MYC and H3K27ac antibodies followed by target-specific PCR and Illumina sequencing was performed 48 h after RNP transfection targeting MDN1 E-box 1 (see Extended Data Fig. 4). Input from crosslinked and sonicated chromatin was used as a control. Precision editing results are shown for all cell lineages harboring sequence tags with exactly two flanking mutations with read count >100 in the input. Log_2_ values for immunoprecipitated (IP) sample/input ratios are shown for each sequence tag pair after calculating the ratio of read counts for mutated vs. original sequence. Black dots represent internal replicates with unique sequence tags, red line indicates median, and the *p*-values are calculated with two-sided Wilcoxon signed rank test, no multiple comparison adjustments (see Supplementary Table 3 for details of statistical parameters). These results support the finding of MDN1 as an important MYC target gene and demonstrate that CGE method combined with ChIP can be used in functional studies in diploid cells.

Because the competitive precision genome editing method showed clear effects on cell proliferation resulting from a mutation of a single MYC binding motif, we set to analyze the direct effects of E-box mutation on MYC binding to the promoter and activation of the promoter as measured by an increase in the active chromatin mark histone 3 lysine 27 acetylation (H3K27ac). For this, we performed ChIP using anti-MYC and anti-H3K27ac antibodies from the HAP1 cells after precision editing. To quantify the editing events, each targeted locus was amplified using polymerase chain reaction (PCR), and the amplicons were Illumina sequenced. We detected fewer antibody-enriched sequences with TATTTA-mutated sequence compared to CACGTG original sequence, demonstrating less MYC binding to the mutated sequences at RPL23, MDN1 and SHMT2 E-boxes, as opposed to the input sample with equal ratios of TATTTA and CACGTG (Fig. 2c). We also observed decrease in H3K27ac at TATTTA-mutated RPL23 and MDN1 E-boxes (Fig. 2c). The markedly lower MYC binding and lower level of activating chromatin mark at these loci indicates that these E-box motifs are biologically active and may contribute to the MYC-dependent expression of the respective genes. However, there were no changes in the level of H3K27ac at the *SHMT2* locus, consistent with the observation that mutation of this E-box had no effect on cell proliferation (Fig. 2b,c). To further test the applicability of the CGE method for studying precise mutations in diploid cells, we performed ChIP using anti-MYC and anti-H3K27ac antibodies after precision editing of the *MDN1* locus in HCT116 colon cancer cells. In agreement with the results from HAP1 cells, we observed less MYC binding and decrease in H3K27ac at alleles harboring TATTTA instead of the native E-box sequence (Extended Data Fig. 6). In conclusion, we identified here several genes that are directly regulated by MYC and demonstrate that mutation of a single MYC binding motif is sufficient for reducing cellular fitness.

**Extended Data Fig. 7:**
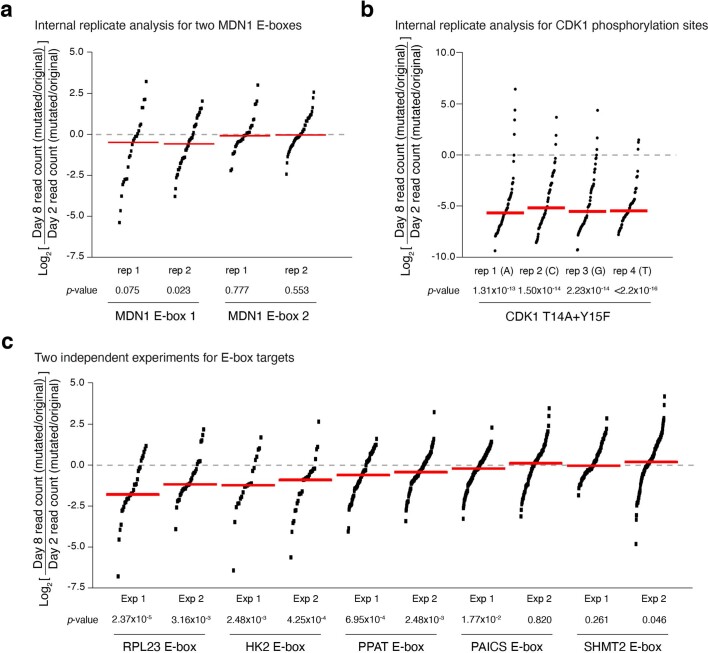
Reproducibility of the CGE experiments. **a, b**, Internal replicate analysis performed by splitting individual cell lineages harboring unique sequence tags to different bins (see Methods). In **a**, cell lineages harboring mutations at the E-box locations of the *MDN1* promoter are binned on the basis of the mutated positions in the 10-bp sequence tag so that the tags having the first flanking mutation at an odd and even positions are separated to two bins, respectively. In **b**, cell lineages harboring CDK1 T14A + Y15F mutations are binned to four groups on the basis of the randomized nucleotides in the sequence tag so that the sequence tags having A, T, G, or C as their first mutated nucleotide are distributed to different groups, respectively. **c**, The fitness effects of E-box mutations from two independent experiments, demonstrating the reproducibility of the results from the CGE method. In panels **a-c**, each dot represents an individual cell lineage harboring a unique barcode. Log_2_ values for read count ratios (day 8/day 2) are shown for each sequence tag pair after calculating the ratio of read counts for mutated vs. original sequence at each time point. Red lines represent the median values; *p*-values from two-sided Wilcoxon signed rank test are shown for each experiment (no multiple comparison adjustments; see Supplementary Table 3 for details of statistical parameters).

**Extended Data Fig. 8:**
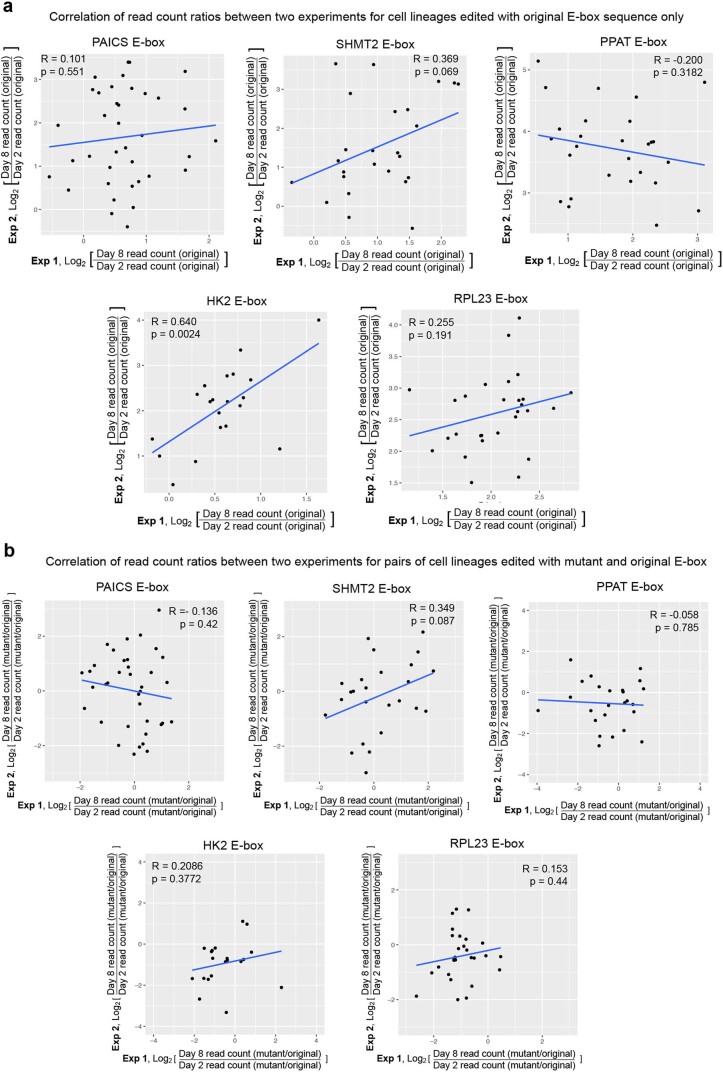
Analysis of the effect of flanking mutations on cellular fitness. **a**, Log_2_ values for read count ratios (day 8/day 2) for cell lineages edited with repair templates harboring the original E-box sequence and a unique sequence tag. **b**, Log_2_ values for read count ratios (day 8/day 2) for cell lineage pairs with a unique sequence tag after calculating the ratio of read counts for mutated vs. original sequence at each time point. In panels **a** and **b**, the results are shown for two independent replicate experiments, showing the sequence tags that were recovered above cut-off (read count >50 on day 2) from both replicates. For PAICS, SHMT2, and PPAT, sequence tags harboring two flanking mutations are shown. For RPL23 and HK2, the recovered sequence tags with two mutations were largely different in the two replicate experiments and thus the log_2_ values for cell lineages harboring one flanking mutations are shown for them.

The large number of individual cell lineages analyzed within one experiment gives the CGE method a high statistical power for measuring phenotypic effects of specific mutations, as shown here for protein phosphorylation sites and MYC binding sites. The sequence tags allow following the growth of cell lineages independently, because the measurement of abundance of each lineage is not dependent on the others within the same culture. The internal replicates also allow splitting the data to internal replicate groups for further statistical analyses (see also ref. ^37^). To demonstrate the robustness of the internal replicate analysis, we grouped the internal replicates into two or four separate groups by binning them based on the mutations within their sequence tags (Methods and ref. ^37^). The internal replicate analysis showed that the medians of the groups are highly similar to each other both for the targeted E-boxes at the *PPAT* and *MDN1* promoters (Fig. 2d and Extended Data Fig. 7a; see also Supplementary Table 3) and for the phosphorylation sites of CDK1 (Extended Data Fig. 7b). To further demonstrate the reproducibility of the results obtained using the CGE method, we performed independent experiments targeting the same E-boxes at the MYC target gene promoters. The results were highly reproducible both for the targets that showed a fitness effect, such as RPL23, HK2 and PPAT, and for the targets that did not, such as PAICS and SHMT2 (Fig. 2d and Extended Data Fig. 7c), indicating the robustness and high statistical power of the CGE method. The replicate experiments also enable studying whether the mutations that generate the sequence tags are silent or near-silent as intended. To this end, the read count ratios between day 8 and day 2 were plotted for the sequence tags that were present in both replicate experiments both for cell lineages that were edited with the original E-box sequence only (Extended Data Fig. 8a) and for the pairs of cell lineages edited with mutant and original sequences harboring identical sequence tags (Extended Data Fig. 8b). Overall, there was no correlation in the read count ratios measured from cell lineages with identical sequence tags between the two replicates, and only one of the targets (HK2) showed statistically significant correlation between the replicates (Extended Data Fig. 8a). These results demonstrate that the CGE method enables measuring the effect elicited by each mutation, but that, overall, the flanking mutations did not contribute to the observed fitness effects or the variation between cell lineages in the assay. The variation between the internal replicates is, thus, likely to reflect different growth rates between lineages as well as different numbers of cells that were transfected with each individual tag. Such variation is inherent to cell-based assays, but our method is robust to the variation and able to precisely measure the biological effect of each mutated target, whereas, if the assay were performed without the sequence tags, the true biological effect could be masked by the variation. It should be noted, however, that internal replicates do not capture day-to-day variation of the experiments, which can, for example, arise from small changes in culture conditions or transfection that affect the growth rate of the cell population. To control for such day-to-day variation, separate independent experiments should be performed (Extended Data Fig. 7c).

Here we show a method for precise analysis of the effect of mutations on cellular phenotype by using CRISPR–Cas9 precision editing combined with lineage-tracing sequence tags and employ it for studying the precise effects of individual TF binding sites and post-translational modifications. Previously, next-generation-sequencing-based methods, such as GUIDE-seq^38^ and Repair-seq^39^, were developed for assessing the off-target DNA cleavage sites and the repair mechanisms of Cas9-induced DNA breaks, respectively. Moreover, random sequence labels have been used for increasing precision and accuracy of CRISPR screens^37^ and DSB-independent base editors for improving the predictability of the Cas9-induced genetic variation in the pooled screens^40,41^. The advantage of our CGE method over these approaches is that both pooled CRISPR screens and high-throughput base editing approaches rely on inferring mutations from the presence of an sgRNA and, thus, require additional validation, whereas the CGE method enables analyzing the mutated loci directly. In a recent saturation mutagenesis screen, a repair template library with single-nucleotide variants (SNVs) targeting the *BRCA1* gene was transfected to target cells along with Cas9 and sgRNA, and targeted gDNA and RNA sequencing was performed to quantify SNV abundances^42^. This method enables distinguishing the edited cells from non-edited ones, providing a powerful method for analyzing the SNVs within coding regions of the target gene studied. Compared to saturation mutagenesis, which is highly effective in analyzing individual genes, CGE is more suitable for dissecting genetic networks, as it can be used to target a large number of genomic loci. Furthermore, in CGE, the genetic barcode is generated by silent or near-silent mutations within the coding and non-coding genomic regions. Thus, CGE is more precise and yields more statistical power to test the effect of particular targeted mutations, enabling a precise assessment of the effect of mutations with subtle phenotypic effects, such as critical targets of protein kinases or critical binding sites of TFs. Our approach of using parallel editing of the target loci with two HDR templates in a single cell culture has two key advantages over previously described genome editing assays (Fig. 2e). First, silent or near-silent mutations that generate sequence tags to HDR templates provide means to discard all confounding information from the next-generation sequencing output of the method. Second, direct comparison of the mutated sequence to the reconstituted native sequence mitigates all the detrimental off-target effects as well as enables lineage tracing of edited clones, thus providing statistical power to the analysis. When measuring allele-specific phenotypes, the method also allows the use of diploid cells for analysis of phenotypes, such as TF binding or RNA expression. We have demonstrated here that the CGE method combined with ChIP can be successfully used for measuring the effect of E-box mutation on MYC binding and H3K27ac also in diploid colon cancer cells. Measuring RNA expression requires that the coding region of a gene of interest harbors a genetic barcode that enables linking the expression measurement to the experimental mutation of the TF binding site. The long-range genome editing for concurrent mutation of the coding region and the TF binding site could be achieved, for example, using recently reported dual prime editing strategies (such as refs. ^43–45^). Measuring more complex phenotypes in diploid cells is also possible, but it requires either prior deletion of one allele from the targeted locus or dilution of the two repair templates by a template that inactivates the wild-type allele in such a way that most cells carry either two inactive alleles or one inactive allele and one targeted allele. This will be easier when targeting coding regions, as failure of targeted repair commonly leads to inactivation of the target gene due to generation of frameshift or deletion alleles by non-homologous end-joining (NHEJ).

The CGE method is particularly useful for studying the effect of small sequence features, such as individual TF binding sites and post-translational modifications, as shown here for MYC binding motifs and phosphorylation sites in the CDK1 and GRB2 proteins, because precision editing is not dependent on finding a highly specific guide sequence precisely overlapping the feature of interest. In addition, the phenotypic impact of such mutations is often milder than that of complete loss of function of the upstream TF, kinase or phosphorylated target. Because the experimental design of the CGE method mitigates the phenotypic effects associated with the genome editing process itself, the method is sensitive enough to detect the subtle effects resulting from mutating TF binding sites and post-translational modifications. Here we identify several MYC binding motifs at the promoters of its target genes that are critical for cellular fitness. The critical target genes represent the major pathways previously associated with MYC function^17^: (1) ribosome biogenesis, including RPL23, a component of 60S large ribosomal subunit, and MDN1, a nuclear chaperone required for maturation and nuclear export of pre-60S ribosome subunit^46^; (2) cellular metabolism, as shown for glycolytic enzyme HK2; as well as (3) nucleotide synthesis, as shown for PPAT involved in de novo purine biosynthesis. However, mutation of the E-box at the *SHMT2* promoter had no effect on cellular fitness in HAP1 cells, although SHMT2 has been previously shown to partially rescue the growth defects of Myc-null fibroblast cells^47^. These results highlight the importance of precise quantitative studies for determining the functional consequence of transcriptional regulatory events on cellular phenotype.

In summary, we report here an advanced method for measuring the phenotypic effects of precise targeted mutations. The method allows controlling for the effect of DNA damage, which is the major confounder in CRISPR-based methods. We also demonstrate the power of the technology by robustly detecting small fitness effects of individual TF binding motifs and single amino acid substitutions. The method is widely applicable and extends the utility of CRISPR–Cas9-mediated genome editing to address important biological questions that have been difficult to address using existing technologies. Using this technology, we identified several target genes whose regulation via canonical E-boxes is responsible for the growth-promoting activity of the universal oncogene MYC.

## Methods

### Genome editing constructs

Each genomic locus was edited by introducing a CRISPR–Cas9-mediated DSB and a locus-specific HDR template library. Guide sequences were designed using CRISPOR^48^ (version 4.99; http://crispor.tefor.net/), giving preference to the protospacers closest to the genomic feature to be edited. The CRISPR RNAs (crRNAs) were obtained from Integrated DNA Technologies (Supplementary Tables 1 and 2). Single-stranded 100-nt DNA molecules were used as HDR templates. For editing E-box sequences, HDR template libraries with two experimental variants were designed for each targeted locus, one with CACGTG sequence for reconstituting the original E-box and another with TATTTA sequence to replace the E-box with a non-functional sequence. In each oligo, the original or mutated E-box was flanked by a 10-nt sequence tag and two 42-nt homology arms complementary to the target strand (Extended Data Fig. 1c). Sequence tags were generated by mutating each of the ten nucleotides with probability of 24%—that is, 8% probability for each of the three non-consensus bases (oligo synthesis using custom hand-mixed bases from Integrated DNA Technologies; Supplementary Table 1). As a negative control, the coding region of the *MYC* gene was targeted with two HDR templates, one reconstituting the original coding sequence and another replacing nucleotides encoding Val-5 and Ser-6 with synonymous codons (GTTAGC > GTAAGT) with a sequence tag created by randomizing the third degenerate position in the two codons flanking the targeted region on both sides (Supplementary Table 1).

For targeting protein phosphorylation sites in the *CDK1* and *GRB2* genes, HDR oligos were designed with 40-nt homology arms and sequence tags by randomizing the degenerate positions of the codons adjacent to the phosphorylation sites (Supplementary Table 2). Experimental variants were the original and mutated sequences of the phosphorylated serine/threonine or tyrosine residues: the sites were abolished by alanine or phenylalanine substitutions or mutated to phosphomimetic glutamate or aspartate residues^23^. Prime editing guide RNAs (pegRNAs) to target CDK1 were designed according to the recommendations from ref. ^33^. Similarly to HDR templates described above, the pegRNA pool introduces a mutation (Y15F) or reconstitutes the original sequence, and, in both cases, the third degenerate position in the codons flanking the targeted region was randomized to create the sequence tags (Supplementary Table 2).

PCR primers for amplifying gDNA at each targeted locus were designed not to have any overlap with the homology arms used in the HDR templates. All custom oligos used for targeting and amplifying the E-box loci and the phosphorylation sites are listed in Supplementary Tables 1 and 2, respectively.

### Cell lines and transfections

HAP1 (C631) and KMB-7 (C628) cell lines were obtained from Horizon Discovery and maintained in low-density cultures in Iscove’s Modified Dulbecco’s Medium (IMDM) with 10% FBS, 2 nM L-glutamine and 1% antibiotics, according to the vendor’s guidelines. The HCT116 cell line (CCL-247) was obtained from the American Type Culture Collection and maintained in McCoy’s 5A (Modified) medium supplemented with 10% FBS and 1% antibiotics, according to the vendor’s guidelines.

CGE experiments measuring cellular fitness were done by transfecting 200,000–400,000 early-passage HAP1 or KBM-7 cells with ribonucleoprotein (RNP) complex together with the HDR template libraries. For sgRNA molecules, equimolar ratios of target-specific crRNAs and ATTO550-tracrRNA (Integrated DNA Technologies) were annealed. RNP complexes used for the transfections were constituted from S.p. HiFi Cas9-protein (Integrated DNA Technologies; 1,000 ng per 200,000 cells) and target-specific sgRNA (250 ng per 200,000 cells) and transfected to cells using CRISPRMAX (Life Technologies), as per the manufacturer’s recommendations, along with HDR template (1:1 mixture of the original and mutant HDR templates) with final concentration of 3 nM. Half of the cells were harvested for gDNA isolation 48 hours after transfection (day 2). The other half was plated for culture on a 10-cm dish, passaged on a T175 flask on day 5 and harvested for gDNA isolation on day 8. For ChIP assays measuring the effect of E-box mutation on MYC occupancy and H3K27 acetylation, 15 million HAP1 cells and 7 million HCT116 cells were transfected for each condition on two 15-cm dishes, scaling up the components of the transfection mix according to the cell numbers. The cells were harvested and chromatin cross-linked 48 hours after transfection.

**Extended Data Fig. 9:**
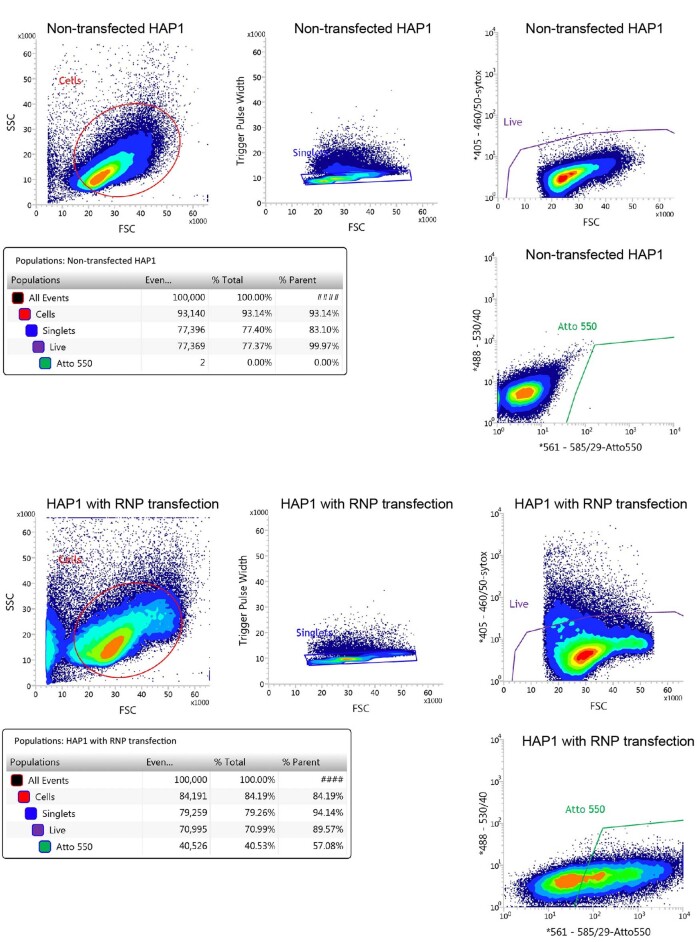
Transfection efficiency of HAP1 cells. Flow cytometry analysis was used for measuring the transfection efficiency of HAP1 cells, since tracrRNA used for generating the RNP complexes for CGE experiment contain ATTO550 fluorochrome. Manual gating was performed using non-transfected control cells (top panel), and similar gates were applied for transfected samples to analyze transfection efficiency (lower panel). Gating strategy from left to right: 1. FSC/SSC: Cells were gated on the main population, excluding clear outliers such as cell debris. 2. FSC/Trigger pulse width: Cells were gated on the main population that represent single cells, excluding the outliers with larger trigger pulse width representing potential duplets. 3. FSC/405 nm (ex. 405 nm, em. 460/50 nm for SYTOX Blue dead cell stain): Cell viability was checked using the SYTOX stain and SYTOX-negative cells were gated to exclude the dead cells with higher fluorescence values. 4. Fluorescence was monitored on two channels: ex. 488 nm, em. 530/40 nm as an extra negative control, and ex. 561 nm, em. 585/29 nm for ATTO550. Gate was set using the non-transfected HAP1 cells so that all cells remained negative for ATTO550. Same gate was maintained to analyze transfected cells to measure the proportion of ATTO550-positive cells. Of note, flow cytometry was used for assessing the transfection efficiency of HAP1 cells, but the cells were not sorted for the CGE experiments.

The transfection efficiency of HAP1 cells was analyzed using flow cytometry, using the ATTO550 fluorochrome within the tracrRNA molecules. Cells transfected with the RNP complex targeting HK2 E-box along with non-transfected control cells were trypsinized 24 hours after transfection, washed once and resuspended in cold PBS, passed through a 35-nm strainer and mixed with SYTOX Blue Dead Cell Stain (Invitrogen), according to the manufacturer’s instructions. The flow cytometry analysis was performed at the HiLife Flow Cytometry Unit, University of Helsinki, Finland, using BD Influx System (USB) and BD FACS software (version 1.2.0.142). The SYTOX stain was excited at 405 nm and ATTO550 at 561 nm, and the gating was set to exclude all dead cells and all non-transfected cells, as detailed in Extended Data Fig. 9.

For prime editing experiments, prime editor 2 was expressed from pCMV-PE2 and pegRNAs from pU6-pegRNA-GG-acceptor plasmids^33^ (Addgene, 132775 and 132777, respectively). Plasmid transfection in HAP1 cells was performed using FuGENE-HD (Promega), according to the manufacturer’s instructions. The rest of the experiment was performed in the same way as the homology-directed editing experiment.

### gDNA isolation and target-specific sequencing

gDNA was isolated using AllPrep DNA/RNA Mini Kit and Blood & Cell Culture DNA Maxi Kit (Qiagen) from day 2 and day 8 samples, respectively, and treated with RNAse A (0.2 μg ul^−1^; Thermo Fisher Scientific) for 2 hours at 37 °C. To eliminate carry-over of single-stranded DNA from the HDR templates, gDNA was treated with exonucleases I and VII (New England Biolabs) in 10 mM Tris-HCl, 50 mM KCl, 1.5 mM MgCl_2_ for 30 minutes at 37 °C, followed by enzyme inactivation for 10 minutes at 95 °C and DNA extraction using phenol:chloroform:isoamyl alcohol (Sigma-Aldrich). Libraries for Illumina sequencing were generated from the gDNA samples in two consecutive PCR reactions using NEBNext High Fidelity Master Mix (New England Biolabs). In PCR1, the edited loci were amplified using target-specific primers with Illumina adaptor flanks (Supplementary Tables 1 and 2) for 20 cycles (using all gDNA material from day 2 and 10 μg of gDNA from day 8 corresponding to 3 million haploid cells as a template) with a maximum of 2.5 μg of gDNA per reaction, followed by DNA purification using 1.5× AMPure XP beads (Beckman Coulter). For the E-box targets, biotinylated primers were used in PCR1 (Supplementary Table 1) for separating the PCR product from the HDR templates. For this, 30% volume of the purified PCR1 products was used for streptavidin capture with M-280 Dynabeads (Thermo Fisher Scientific), according to the manufacturer’s protocol. Prime editing samples are not affected by the presence of HDR template, and, thus, they were prepared without the exonuclease I/VII treatment and affinity purification of biotinylated PCR1 products. In PCR2, sequencing-ready libraries were generated by amplifying the products from PCR1 for eight cycles using NEBNext High Fidelity Master Mix and Illumina Universal and Index primers (E7335S, New England Biolabs). Four and 12 parallel reactions on M-280 beads were set for day 2 and day 8 samples, respectively. PCR2 products were purified using 0.9× AMPure XP beads. The correct library sizes (corresponding to each PCR product) were confirmed using TapeStation 4200 (DNA D5000 High Sensitivity tape; Agilent), and library quantification was performed using KAPA Library Quanitification Kit for Illumina platforms using LightCycler 480 (Roche), according to the manufacturer’s recommendations. Libraries representing >8 distinct targeted loci were pooled to ensure the necessary sequence complexity for amplicon sequencing and sequenced for 150 cycles on NovaSeq 6000, HiSeq 4000 and NextSeq 500 platforms (Illumina) with 1% PhiX. Sequencing depth for each sample is provided in Supplementary Table 4.

### Pre-processing of the sequences from precision editing experiments

Sequencing reads were demultiplexed using bcl2fastq (version 2.20), and FastQC analysis (version 0.11.9; http://www.bioinformatics.babraham.ac.uk/projects/fastqc/) was performed for read numbers and quality. Reads were assigned to each genomic target by fetching the sequences with a perfect match to the first 20 nucleotides of each locus-specific PCR product from the fastq.gz-files using zgrep (version 1.10). Then, grep (version 3.4) with -o command was used for extracting 16-nt parts of the reads that contain either original sequence (such as CACGTG) or mutated sequence (such as TATTTA) and any five nucleotides flanking them on both sides. The approximate string-matching program agrep^49^ (version 3.0) was then used for finding the patterns with exact matches to original and mutant E-box with, at most, two flanking mutations, using high cost values for insertions and deletions (-I5 -D5) so that the actual sequence tags generated by substitutions are printed in the output. For mutations at the coding regions, the exact matches for each specific variation of the repair templates were counted directly from the fastq.gz files using zgrep -o (version 1.10) and wc command.

### Analysis of the fitness effects

Reads for each unique sequence tag were counted (uniq -c; version 8.30), and a pseudocount of +1 was added to each value to avoid zeros in subsequent calculations. For experiments targeting E-boxes, all cell lineages harboring sequence tags with read count >50 in both day 2 samples (mutant and control) were included in the analyses. In Fig. 1d, the results from all 30 sequence tags with one flanking mutation are shown to demonstrate the power of the method in tracing the growth of individual cell lineages over time. To further increase the robustness of the analysis, the sequence tags with two flanking mutations were used in the analyses for Fig. 2b–d and Extended Data Figs 5–7: because sequences with wild-type E-box and only one flanking mutation could have resulted from errors in PCR or Illumina sequencing, all the sequence tags with only one mutation were excluded from the analyses to avoid such artifacts, although such sequences would represent only a minority of the data,. The sequence tags with three mutations were not included in the analyses, because, for most targets, their read counts did not meet the inclusion criteria at the sequencing depth used in this study. This is because the oligos with fewer flanking mutations are overrepresented in the pool of HDR oligoes due to the mutation strategy used in their synthesis (Extended Data Fig. 1), and, thus, the read counts are inversely correlated with the number of flanking mutations, as shown in Fig. 1c. For experiments targeting protein phosphorylation sites, all the sequence tags with read count >20 for GRB2 experiments and >5 for CDK1 experiments were included in the analyses due to lower number of reads from the edited cells in these experiments.

To analyze the effect of each mutation on cellular fitness, the ratio of cells harboring mutated and original sequence features was compared at each timepoint. To eliminate the potential effect of near-silent flanking mutations on cellular fitness, the sequence tags with the same flanking mutations were compared to each other in the analysis. In Fig. 2 and in Extended Data Figs. 3, 5–7 and 8b, the results are presented as log_2_(fold change) as follows: log_2_[day 8 read count (mutated/original)/day 2 read count (mutated/original)]. Each sequence tag pair represents an individual cell lineage or lineage pool (if multiple cells have been transfected with similar oligo). Two-sided Wilcoxon signed-rank test was used for testing whether the median of log_2_(fold change) is unequal to zero. Each sample was measured only once (not repeatedly); no adjustment for multiple comparisons was used. All statistical parameters, including sample size, median, *P* value, standard deviation and confidence intervals, are shown for each target in Supplementary Table 3.

### Estimating the editing efficiency

For calculating the proportion of the cells that have undergone precision editing using the HDR templates, sequence tags with, at most, five flanking mutations were extracted from the day 2 and ChIP input samples for the E-box targets using agrep -5 -D5 -I5, as described above. Based on the mutation strategy used for generating the sequence tags, sequences with 0–5 mutations represent 98.4% of all the sequences in the repair template libraries (Extended Data Fig. 1a). The sum of read counts for all sequence tags with original and mutated sequence was calculated for each target. Then, the reads with exact match to the original sequence without any flanking mutations were considered as wild-type unedited cells and subtracted from the total read count from above agrep analysis (although the chosen mutation strategy also generates wild-type-like sequences). The sequences that did not match the wild-type sequence nor the expected HDR templates were considered to be Cas9 edits resulting from NHEJ. Of note, NHEJ events can also produce sequences similar to HDR templates with the original E-box and one flanking mutation, and sequencing errors and PCR artifacts may contribute to the reads assigned to the NHEJ category or to sequences with wild-type E-box and only one flanking mutation. However, the proportions of reads matching to the repair templates were consistent between replicate experiments, suggesting that the observed mutation patterns originate largely from the true genome editing events and not random artifacts. The artifacts might, however, result in overestimation of the NHEJ reads, especially for the targets with low editing efficiency. The proportions of wild-type sequences, NHEJ edits and reads matching to the repair templates are listed in Supplementary Table 4.

All recovered sequence tags using agrep -5 -D5 -I5 command from ChIP input sample for *SHMT2* locus were also used for analyzing the distribution of flanking mutations on day 2, as shown in Extended Data Fig. 2a. Sequence logos were generated using WebLogo^50^ (version 2.8.2; https://weblogo.berkeley.edu/logo.cgi) by aligning all sequence tags with at least one flanking mutation observed in the sample. The sequence logo for the expected distribution of flanking mutations for the *SHMT2* locus shown in Fig. 1a and in Extended Data Fig. 2a was generated based on the mutation probability for each mutation—that is, 8% for each non-consensus base flanking the E-box or the TATTTA sequence.

### Replicate analysis

Internal replicate analysis was performed by grouping the cell lineages into two or four groups based on the mutations within their sequence tags. For binning the CDK1 T14A + Y15F lineages, the exact mutation of the first randomized nucleotide (Supplementary Table 2; the underlined N closest to the 5′ end of the HDR oligo) was used for separating the sequence tags into four groups shown in Extended Data Fig. 7b with A, C, T or G at the first position. For E-box targets, the binning was based on the position of the mutations within the sequence tags generated by mutating each of the ten nucleotides flanking the sequence of interest with the probability of 24%. Each of the potentially mutated positions is marked by NX:76080808 notation in the repair template oligos listed in Supplementary Table 1. For the purposes of the binning, the potentially mutated flanking positions were numbered from 1 to 10 in the 5′–3′ direction, and lineages were separated into two groups based on the first non-consensus nucleotide detected in each sequence tag (that is, the flanking mutation closest to the 5′ end of the repair template oligo; Supplementary Table 1). The sequence tags with their first flanking mutation at odd position (1, 3, 5, 7 or 9) or even position (2, 4, 6 or 8) were grouped together, respectively. The analysis of read count ratios and calculation of median and *P* values for internal replicate groups was done as described for the analysis of fitness effects.

For E-boxes at the promoters of *RPL23*, *HK2*, *PPAT*, *PAICS* and *SHMT2* genes, two independent experiments were performed on separate days from different batches of cells, and all experimental and data analysis steps were performed independently for each experiment. The replicates were also used for analyzing the potential effects of the flanking mutations on the cellular fitness from the sequence tags with read count >50 on day 2 in both replicates (Extended Data Fig. 8). Correlation coefficient (R) and *P* values were calculated using Pearson’s product moment correlation. log_2_(fold change) for read count ratios (day 8/day 2) for cell lineages edited with repair templates harboring only the original E-box sequence as well as pairs of cell lineages edited with mutated and original sequence and the similar flanking mutations are shown for sequence tags with two flanking mutations for PAICS, SHMT2 and PPAT and sequence tags with one flanking mutation for RPL23 and HK2.

### ChIP with target-specific sequencing and ChIP-seq

Wild-type HAP1 and genome-edited HAP1 and HCT116 cells were cross-linked with 1% formaldehyde 48 hours after RNP transfection, and chromatin samples were prepared as described previously^51^. Chromatin was sonicated to an average fragment size of 500 bp using a micro-tip sonicator (Misonix) and used for immunoprecipitation (IP) with antibody-coupled Dynabeads (Thermo Fisher Scientific) for MYC, H3K27ac and normal rabbit IgG (Millipore, 06-340; Abcam, ab4729; and Santa Cruz Biotechnology, sc-2027, respectively, 5 µg of antibody/IP). The amount of chromatin corresponding to 10 million wild-type cells and 20 million transfected cells was used for each IP. After overnight incubation, 5× washes with LiCl buffer and reverse cross-linking was performed as described in ref. ^51^, followed by DNA purification using phenol:chloroform:isoamyl alcohol and ethanol precipitation.

All immunoprecipitated DNA isolated from transfected cells was amplified for 30 cycles in two reactions using similar PCR1 conditions and primers as described above for gDNA samples. In addition, 10 μg of input DNA from each transfected condition was amplified in four parallel reactions. PCR1 products were purified using 1.5× AMPure XP beads, and 20% of purified DNA was used as a template in PCR2 for eight cycles with Illumina primers as above. Final libraries were purified using 0.9× AMPure XP beads. Quality control and pooling were performed as described above for gDNA libraries, and the pooled libraries were sequenced for 150 cycles on NovaSeq 6000 (Illumina) with 1% PhiX. Data were analyzed essentially as described for fitness experiments: after excluding the reads originating from wild-type cells, a pseudocount +1 was added to the read count values; the read count ratios between mutated and original sequences were calculated for each condition; and log_2_(fold change) between each IP and respective input sample was calculated. Only sequence tags with read counts >100 in the input sample were included in the analyses. Two-sided Wilcoxon signed-rank test was used for testing whether the median of log_2_(fold change) values is unequal to zero.

Wild-type HAP1 samples were used for standard ChIP-seq library preparation with NEBNext Ultra II DNA Library Prep Kit (New England Biolabs), followed by sequencing on NovaSeq 6000. The reads were aligned to human genome (hg19) using bowtie2 (ref. ^52^) (version 2.2.4), and peaks were called using MACS2 (ref. ^53^) (version 2.1.1) with default narrow peak parameters against input or normal IgG for MYC and H3K27ac, respectively. The bedgraph files were used for genome browser snapshots. For colon cancer cell lines GP5d, LoVo and COLO320DM, previously published ChIP-nexus datasets from ref. ^34^ (EGAD00001004099) were used. In the genome browser snapshots, the traces from BAM coverage files are shown.

### Chromatin accessibility and gene expression analysis

ATAC-seq for chromatin accessibility was performed from 50,000 HAP1 cells, as previously described^54^. In brief, cells were washed with ice-cold PBS, lysed in 50 μl of lysis buffer for 10 minutes on ice and treated with Tn5 transposase in 2× tagmentation buffer (Illumina) for 30 minutes at 37 °C. DNA was purified using MinElute PCR Purification Kit (Qiagen) and prepared for sequencing using Nextera library preparation kit (Illumina) by five cycles of PCR amplification. The library was sequenced on NovaSeq 6000 for 2 × 50 cycles, and the paired-end data were analyzed using an in-house pipeline comprising the following software: TrimGalore (version 0.4.3; https://www.bioinformatics.babraham.ac.uk/projects/trim_galore/), BWA aligner^55^ (version 0.7.15), Picard (version 2.9.2; http://broadinstitute.github.io/picard) and broad-peak calling by MACS2 (ref. ^53^) (version 2.1.1), as described in ref. ^35^. For GP5d cells, the ATAC-seq data from ref. ^35^ (GSE180158) was used. In the genome browser snapshots, the traces from BAM coverage files are shown.

For gene expression analysis, previously published RNA-seq data from ref. ^34^ (EGAD00001004098) were used. The datasets for MYC silencing using siRNA (siMYC), and respective control samples transfected with non-targeting siRNAs (siNon-target) for GP5d and LoVo cells were re-analyzed by aligning the reads from FASTQ files to human genome (hg19) using tophat2 (ref. ^56^) (version 2.0.13) and by analyzing the differentially expressed genes between siMYC and siNon-target samples using cuffdiff^57^ (version 2.2.1) using default parameters for first-strand library type.

### Reporting summary

Further information on research design is available in the Nature Research Reporting Summary linked to this article.

## Online content

Any methods, additional references, Nature Research reporting summaries, source data, extended data, supplementary information, acknowledgements, peer review information; details of author contributions and competing interests; and statements of data and code availability are available at 10.1038/s41587-022-01444-6.

## Supplementary information

Supplementary Information Supplementary Tables 1–4.

Reporting Summary

Supplementary Table 5 Supplementary Table 5. Read counts for individual cell lineages for the results shown in Fig. 2b.

## Data Availability

All next-generation sequencing data generated from the CGE experiments as well as the HAP1 ATAC-seq data are available in the European Nucleotide Archive (ENA) under accession number PRJEB52351 (ref. ^58^). ChIP-seq data generated in this study are available under Gene Expression Omnibus accession number GSE206080 (ref. ^59^). Human genome sequence was used from the Genome Reference Consortium Human Build 37 (GRCh37; hg19) under accession number GCA_000001405. Previously published datasets for colon cancer cells were used as follows: RNA sequencing from EGAD00001004098 (ref. ^60^), ATAC-seq from GSE180158 (ref. ^61^) and ChIP-nexus from EGAD00001004099 (ref. ^62^).
