# Supplementary material for: A competitive precision CRISPR method to identify the fitness effects of transcription factor binding sites
Source: Nat Biotechnol. 2022 Sep 26;41(2):197–203. doi: 10.1038/s41587-022-01444-6 (PMC9931575; doi:10.1038/s41587-022-01444-6)
Supplement: Supplementary file 1 — Supplementary Tables 1–4. [file 41587_2022_1444_MOESM1_ESM.pdf]

nature portfolio

https://doi.org/10.1038/s41587-022-01444-6

Supplementary information

A competitive precision CRISPR method to identify the fitness effects of transcription factor binding sites

In the format provided by the authors and unedited

Supplementary Information

A competitive precision CRISPR method to identify the fitness effects of transcription factor binding sites

Päivi Pihlajamaa[1,2], Otto Kauko[1,2,3], Biswajyoti Sahu[1,4], Teemu Kivioja[1,2] and Jussi Taipale[1,2,5]*

1. *Applied Tumor Genomics Research Program, Faculty of Medicine, University of Helsinki, Helsinki, Finland*
2. *Department of Biochemistry, University of Cambridge, Cambridge, United Kingdom*
3. *Turku Bioscience, University of Turku, Turku, Finland*
4. *Medicum, Faculty of Medicine, University of Helsinki, Helsinki, Finland*
5. *Department of Medical Biochemistry and Biophysics, Karolinska Institute, Stockholm, Sweden*

* *Corresponding author: Jussi Taipale (ajt208@cam.ac.uk)*

Supplementary Information includes:

Supplementary Tables 1-4

Supplementary Table 5 (as a separate excel-file)

Supplementary Table 1. Custom oligos used for targeting MYC binding sites. The sequence tags were generated for HDR oligos with defined ratios of wild-type nucleotide (probability 76%) and mutations (probability 24%; each of the three non-wild type nucleotides 8%). The ratios for different nucleotides in brackets are in the following order: A, C, G, T as per the specifications for hand-mixed bases from Integrated DNA Technologies. For each genomic target, the two HDR templates (original and mutated) are transfected simultaneously.

Name	Sequence 5'-3'
**HDR templates**	
SHMT2_Original	CAGCACCGGGTCCGCGGGGCATCCGGGGCTGTCCTCTCCCCT (N1:76080808)(N1) (N2:08080876)(N3:08087608)(N4:08760808) CACGTG (N1)(N1)(N1)(N2)(N3) GGCGGTCACGAGGCCCTGGGGCGTCTGATCGCCAGAATGAGG
SHMT2_Mutated	CAGCACCGGGTCCGCGGGGCATCCGGGGCTGTCCTCTCCCCT (N1:76080808)(N1) (N2:08080876)(N3:08087608)(N4:08760808) TAAATA (N1)(N1)(N1)(N2)(N3) GGCGGTCACGAGGCCCTGGGGCGTCTGATCGCCAGAATGAGG
PAICS_Original	TACTCCCATTACGCTACTGTTGGGTGCTGGAAAATAACGGAG (N1:76080808)(N2:08080876) (N2)(N2)(N4:08760808) CACGTG (N4)(N1)(N3:08087608)(N4)(N1) AATACAGCTTTCCAGGCAGCTCTTAGGGGAAGGCTAGCTCCA
PAICS_Mutated	TACTCCCATTACGCTACTGTTGGGTGCTGGAAAATAACGGAG (N1:76080808)(N2:08080876) (N2)(N2)(N4:08760808) TAAATA (N4)(N1)(N3:08087608)(N4)(N1) AATACAGCTTTCCAGGCAGCTCTTAGGGGAAGGCTAGCTCCA
PPAT_Original	TGGCAGCTGGTTGGTGCTTACACCTTGGCCGCAGCGGCAGGT (N4:08760808)(N4) (N2:08080876)(N2)(N4) CACGTG (N4)(N2)(N2)(N2)(N4) GGCGGCGACATGGAGCTGGAGGAGTTGGGGATCCGAGAGGAA
PPAT_Mutated	TGGCAGCTGGTTGGTGCTTACACCTTGGCCGCAGCGGCAGGT (N4:08760808)(N4) (N2:08080876)(N2)(N4) TAAATA (N4)(N2)(N2)(N2)(N4) GGCGGCGACATGGAGCTGGAGGAGTTGGGGATCCGAGAGGAA
HK2_Original	TCCACGTGCTCTCGCCGGAACTCGCGCTGCAGAAGGGGGCTC (N4:08760808)(N4) (N2:08080876)(N4)(N4) CACGTG (N3:08087608)(N3)(N4)(N3)(N4) AGCAATCCCTGACTACCTGCGGGGTGACCCGCCCCTCCAGTA
HK2_Mutated	TCCACGTGCTCTCGCCGGAACTCGCGCTGCAGAAGGGGGCTC (N4:08760808)(N4) (N2:08080876)(N4)(N4) TAAATA (N3:08087608)(N3)(N4)(N3)(N4) AGCAATCCCTGACTACCTGCGGGGTGACCCGCCCCTCCAGTA
RPL23_Original	TAATAAGGCAGCGCCCAGAGGCGGAAGAGGCCGGTTTTTGCT (N4:08760808)(N4) (N3:08087608)(N3)(N4) CACGTG (N1:76080808)(N3)(N3)(N1)(N3) GGTGGGCGGGGCGTTAAAGTTCATATCCCAGTGTCCTTTGAA
RPL23_Mutated	TAATAAGGCAGCGCCCAGAGGCGGAAGAGGCCGGTTTTTGCT (N4:08760808)(N4) (N3:08087608)(N3)(N4) TAAATA (N1:76080808)(N3)(N3)(N1)(N3) GGTGGGCGGGGCGTTAAAGTTCATATCCCAGTGTCCTTTGAA
MDN1_E-box-1 Original	CAGCGTAGCCGTCCCGAGCTGATGACGTCAAACGCCGTGTGC (N2:08080876)(N4:08760808) (N1:76080808)(N4)(N4) CACGTG (N2)(N3:08087608)(N3)(N2)(N3) CCCCTCTCCCGGTGTAGGCGCTGGAGCTGAGGACGCCTTTCC
MDN1E_E-box-1 Mutated	CAGCGTAGCCGTCCCGAGCTGATGACGTCAAACGCCGTGTGC (N2:08080876)(N4:08760808) (N1:76080808)(N4)(N4) TAAATA (N2)(N3:08087608)(N3)(N2)(N3) CCCCTCTCCCGGTGTAGGCGCTGGAGCTGAGGACGCCTTTCC
MDN1_E-box-2 Original	GCCCCCTCTTTCCTCCCCGGCCTAACGCACTCTTCTTTCTGT (N4:08760808)(N4)(N4) (N1:76080808)(N4) CACGTG (N4)(N2:08080876)(N3:08087608)(N2)(N3) TTCGCGAGTCCCCATCACTCCTGCATCCAAACCGACTAGATT
MDN1_E-box-2 Mutated	GCCCCCTCTTTCCTCCCCGGCCTAACGCACTCTTCTTTCTGT (N4:08760808)(N4)(N4) (N1:76080808)(N4) TAAATA (N4)(N2:08080876)(N3:08087608)(N2)(N3) TTCGCGAGTCCCCATCACTCCTGCATCCAAACCGACTAGATT
Neg_Ctrl_Original	TACGGCTGCACCGAGTCGTAGTCGAGGTCATAGTTCCTGTTNGTNAAGCTAACNTTNAGGGGCATCGTCGCGGGAGGCTGCTGGAGCGGGGCACACAAAG
Neg_Ctrl_Mutated	TACGGCTGCACCGAGTCGTAGTCGAGGTCATAGTTCCTGTTNGTNAAACTTACNTTNAGGGGCATCGTCGCGGGAGGCTGCTGGAGCGGGGCACACAAAG
**Protospacer sequences**	
SHMT2_crRNA	TCGTGACCGCCCATTTCACG
PAICS_crRNA	AAAGCTGTATTTGCTGCACG
PPAT_crRNA	ATGTCGCCGCCGAAAGCACG
HK2_crRNA	CAGGGATTGCTGCGCCCACG
RPL23_crRNA	CCCCGCCCACCCTCCTCACG
MDN1_E-box-1 crRNA	CGGGAGAGGGGCACCACACG
MDN1_E-box-2 crRNA	GGACTCGCGAACACAGCACG
Neg_Ctrl_crRNA	TGTTGGTGAAGCTAACGTTG

**Target-specific primers for gDNA amplification**	
SHMT2_gDNA_FP	/5Biosg/ACACGACGCTCTTCCGATCTCAGGGAGCGGACGTGTAAC
SHMT2_gDNA_RP	GACGTGTGCTCTTCCGATCTTGAGACCAGGCCGAAAACTC
PAICS_gDNA_FP	/5Biosg/ACACGACGCTCTTCCGATCTCTCCGAGTCCACCAACGAG
PAICS_gDNA_RP	GACGTGTGCTCTTCCGATCTTACCAAGTCTTGACGGCCTG
PPAT_gDNA_FP	/5Biosg/ACACGACGCTCTTCCGATCTTCCCAGAGTGATCACATGCG
PPAT_gDNA_RP	GACGTGTGCTCTTCCGATCTCTTCTGAGCTCGACGGGC
HK2_gDNA_FP	/5Biosg/ACACGACGCTCTTCCGATCTCCTCCCTCCCTGAGCTCC
HK2_gDNA_RP	GACGTGTGCTCTTCCGATCTGAACCGCTCGTCTCCTACAC
RPL23_gDNA_FP	/5Biosg/ACACGACGCTCTTCCGATCTGCTTCGACATCTTGAACGCC
RPL23_gDNA_RP	GACGTGTGCTCTTCCGATCTTGCGCTTCTACCTCAACTCC
MDN1_E-box-1 gDNA_FP	/5Biosg/ACACGACGCTCTTCCGATCTCACCTGCGCTCCCTACTTC
MDN1_E-box-1 gDNA_RP	GACGTGTGCTCTTCCGATCTCTCCTGCATCCAAACCGACT
MDN1_E-box-2 gDNA_FP	/5Biosg/ACACGACGCTCTTCCGATCTCTTCGCTGTCCCGCCTTTTC
MDN1_E-box-2 gDNA_RP	GACGTGTGCTCTTCCGATCTTCTTTCCGTGTGTTAAGGCAGA
Neg_Ctrl_gDNA_FP	/5Biosg/ACACGACGCTCTTCCGATCTCCGCACCAAGACCCCTTTAA
Neg_Ctrl_gDNA_RP	GACGTGTGCTCTTCCGATCTTCCTCCTCGTCGCAGTAGAA

Supplementary Table 2. Custom oligos used for targeting protein phosphorylation sites. Sequence tags were generated by randomizing the third degenerate position in the codons flanking the region of interest. For CDK1 T14+Y15, the two separate HDR templates (original and mutated) were designed, and in the experiments, they were transfected simultaneously. For all the other mutations at phosphorylation sites, randomized template generates WT, non-phosphorylatable, and phosphomimetic edits. Prime editing of CDK1 Y15 was analyzed with the same target-specific gDNA primers as CDK1 T14+Y15 HDR edits.

**Name**	**Sequence 5′-3′**
**HDR templates**	
CDK1_T161	TGGCTGATTTTGGCCTTGCCAGAGCTTTTGGAATACCTAT**H**AG**R**GT**N**TA**YRM**ACA**Y**GAGGCAAGTGGAATAGTGGTTTTTGATGGCTTTTGAATGT
CDK1_T14_Y15 Original	TGGGGTGTGTCACACAGCATATTATTTACTTTGTTTCAGG**N**ACCTATGG**N**GT**N**GT**N**TATAAGGGTAGACACAAAACTACAGGTCAAGTGGTAGCCA
CDK1_T14A_Y15F Mutated	TGGGGTGTGTCACACAGCATATTATTTACTTTGTTTCAGG**NG**CCT**T**TGG**N**GT**N**GT**N**TATAAGGGTAGACACAAAACTACAGGTCAAGTGGTAGCCA
GRB2_Y160	GGAATGCAATGTGGGTTTTTCCTGCTCCTGTTTTGCAGCA**R**CC**N**AC**NKW**CGT**N**CA**R**GCCCTCTTTGACTTTGATCCCCAGGAGGATGGAGAGCTGG
GRB2_Y209	GGTGGAAAGGAGCTTGCCACGGGCAGACCGGCATGTTTCC**N**CG**N**AA**YKW**TGT**N**AC**N**CCCGTGAACCGGAACGTCTAAGAGTCAAGAAGCAATTATT
**Protospacer sequences**	
CDK1_T161	TATCAGAGTATATACACATG
CDK1_T14_Y15	ACCCTTATACACAACTCCAT
GRB2_Y160	AAGAGGGCCTGGACGTATGT
GRB2_Y209	ACGGGGGTGACATAATTGCG
**pegRNA for prime editing**	
CDK1_Y15	ACCCTTATACACAACTCCATGTTTTAGAGCTAGAAATAGCAAGTTAAAATAAGGCTAGTCCGTTATCAACTTGAAAAAGTGGCACCGAGTCGGTGCTTGTTTCAGG**N**AC**NKWY**GGAGTTGTGTATAA
**Target-specific primers for gDNA amplification**	
CDK1_T161_FP	ACACGACGCTCTTCCGATCTTCTCTTGATTGATGACAAAGGAAC
CDK1_T161_RP	GACGTGTGCTCTTCCGATCTAATTGATACTGCCACAGAGTAG
CDK1_T14+Y15 FP	ACACGACGCTCTTCCGATCTAGATCTTTAGTTTGTGGGGTGTG
CDK1_T14+Y15 RP	GACGTGTGCTCTTCCGATCTGGATGACGAAGTTCCTTTAATAGAG
GRB2_Y160_FP	ACACGACGCTCTTCCGATCTATCTGGCCTCGGAGGTG
GRB2_Y160_RP	GACGTGTGCTCTTCCGATCTAAGCTCCTTTCCACCAGTTG
GRB2_Y209_FP	ACACGACGCTCTTCCGATCTATGGAGAGCTGGGCTTC
GRB2_Y209_RP	GACGTGTGCTCTTCCGATCTGCAGCTTGTGGGTTTAATTC

Supplementary Table 3. Statistical parameters for the analyzed targets. The number of distinct sequence tags used in each analysis is represented by *n*. Two-sided Wilcoxon signed rank test was used for testing whether the median of $\log_2$ (fold change) is unequal to zero; median, *p*-value, 95% confidence interval (CI), and standard deviation (SD) is shown for each target. Fold change = day 8 read count (mutated / original sequence) / day 2 read count (mutated / original sequence).

Sample ID	Figure	*n*	Median	*p*-value	95% CI Min	95% CI Max	SD
RPL23 E-box exp 1	Fig. 2b<br>ED-Fig. 7c	30	-1.68	2.37×10^{-5}	-2.0106	-0.8117	1.7
RPL23 E-box exp 2	ED-Fig. 7c	32	-1.09	3.16×10^{-3}	-1.2689	-0.3843	1.3
HK2 E-box exp 1	Fig. 2b<br>ED-Fig. 7c	21	-1.19	2.48×10^{-3}	-1.7882	-0.3650	1.7
HK2 E-box exp 2	ED-Fig. 7c	28	-0.81	4.25×10^{-4}	-1.8120	-0.5451	1.7
PPAT E-box exp 1	Fig. 2b<br>Fig. 2d<br>ED-Fig. 7c	65	-0.55	6.95×10^{-4}	-0.8967	-0.2593	1.3
PPAT E-box exp 1 internal rep 1	Fig. 2d	35	-0.67	6.65×10^{-3}	-1.0893	-0.1654	1.3
PPAT E-box exp 1 internal rep 2	Fig. 2d	30	-0.41	3.84×10^{-2}	-1.0299	-0.0373	1.3
PPAT E-box exp 2	Fig. 2b<br>Fig. 2d<br>ED-Fig. 7c	90	-0.36	2.48×10^{-3}	-0.5890	-0.1249	1.1
MDN1 E-box 1	Fig. 2b	68	-0.50	5.09×10^{-3}	-1.0654	-0.1888	1.8
MDN1 E-box internal rep 1	ED-Fig. 7a	26	-0.45	7.52×10^{-2}	-1.6679	0.1079	2.2
MDN1 E-box internal rep 2	ED-Fig. 7a	42	-0.54	2.33×10^{-2}	-1.0256	-0.0738	1.5
MDN1 E-box 2	ED-Fig. 5	77	-0.04	0.7878	-0.1710	0.2885	1.0
MDN1 E-box 2 internal rep 1	ED-Fig. 7a	30	-0.05	0.7766	-0.3694	0.3560	1.1
MDN1 E-box 2 internal rep 2	ED-Fig. 7a	47	-0.01	0.5533	-0.1875	0.4104	1.0
PAICS E-box exp 1	Fig. 2b	130	-0.21	1.77×10^{-2}	-0.4127	-0.0350	1.0
PAICS E-box exp 2	Fig. 2b<br>ED-Fig. 7c	72	0.18	0.8202	-0.3080	0.3696	1.3
SHMT2 E-box exp 1	Fig. 2b	70	-0.02	0.2612	-0.1057	0.4315	1.0
SHMT2 E-box exp 2	Fig. 2b<br>ED-Fig. 7c	103	0.26	4.60×10^{-2}	0.0043	0.5815	1.5
MYC negative control	Fig. 2b	55	-0.17	0.4584	-0.6727	0.2555	1.7
HAP1 MDN1 MYC ChIP	Fig. 2c	31	-2.90	3.18×10^{-5}	-5.1616	-2.0421	3.9
HAP1 MDN1 H3K27ac ChIP	Fig. 2c	31	-3.17	1.10×10^{-2}	-4.5774	-0.7608	4.8
HAP1 RPL23 MYC ChIP	Fig. 2c	78	-4.98	9.17×10^{-13}	-5.0234	-3.4848	3.1
HAP1 RPL23 H3K27ac ChIP	Fig. 2c	78	-1.18	4.15×10^{-5}	-2.4269	-0.8121	3.2
HAP1 SHMT2 MYC ChIP	Fig. 2c	238	-2.23	$< 2.2 \times 10^{-16}$	-2.9670	-2.0835	4.2
HAP1 SHMT2 H3K27ac ChIP	Fig. 2c	238	0.04	0.7406	-0.1484	0.2218	1.4
HCT116 MDN1 MYC ChIP	ED-Fig. 6	147	-2.84	$< 2.2 \times 10^{-16}$	-3.7315	-2.5891	3.2
HCT116 MDN1 H3K27ac ChIP	ED-Fig. 6	147	-1.49	2.77×10^{-5}	-2.3773	-0.9257	4.4
CDK1 T161A	Fig. 2a	43	-1.32	2.92×10^{-3}	-2.1478	-0.6378	4.0
CDK1 T161E	Fig. 2a	54	-0.42	0.7055	-1.0089	1.0658	3.9
CDK1 T14A/Y15F	Fig. 2a	239	-5.37	$< 2.2 \times 10^{-16}$	-5.3777	-4.7415	2.6
CDK1 T14A/Y15F internal rep 1 (A)	ED-Fig. 7b	62	-5.57	1.31×10^{-13}	-5.8908	-4.7475	3.0
CDK1 T14A/Y15F internal rep 2 (C)	ED-Fig. 7b	59	-5.07	1.50×10^{-14}	-5.4014	-3.9392	2.7

CDK1 T14A/Y15F internal rep 3 (G)	ED-Fig. 7b	58	-5.42	2.23×10^{-14}	-5.7111	-4.1098	2.8
CDK1 T14A/Y15F internal rep 4 (T)	ED-Fig. 7b	60	-5.37	$< 2.2 \times 10^{-16}$	-5.6300	-4.6945	2.0
CDK1-Y15F Prime editing	Fig. 2a	19	-1.75	2.89×10^{-2}	-3.2424	-0.2565	3.0
GRB2 Y160F HAP1	ED-Fig. 3	253	0.10	0.1039	-0.0249	0.2448	1.2
GRB2 Y160D HAP1	ED-Fig. 3	255	-0.06	0.7479	-0.1541	0.1128	1.2
GRB2 Y209F HAP1	ED-Fig. 3	126	0.26	0.4036	-0.2498	0.5860	2.6
GRB2 Y209D HAP1	ED-Fig. 3	98	-0.29	9.81×10^{-2}	-1.2000	0.1002	3.1
GRB2 Y160F KBM7	ED-Fig. 3	47	0.11	0.7412	-0.8142	1.1861	3.5
GRB2 Y160D KBM7	ED-Fig. 3	42	-1.26	1.53×10^{-4}	-2.2856	-0.7856	2.6
GRB2 Y209F KBM7	ED-Fig. 3	51	-0.76	8.88×10^{-5}	-2.1049	-0.6037	2.1
GRB2 Y209D KBM7	ED-Fig. 3	35	-0.70	1.28×10^{-2}	-1.5476	-0.1703	1.9

Supplementary Table 4. Read counts and estimates of editing efficiency. Editing efficiency has been estimated from the day 2 sample (fitness effect experiments) or input sample (ChIP experiments) by analyzing the number of reads that match to the expected sequence tags. The sequences that did not match the wild-type sequence nor the expected HDR templates were considered to be Cas9-edits resulting from non-homologous end-joining (NHEJ; see **Methods** for more details about assigning the reads to different categories).

Fitness experiments for E-box targets

Experiment	Total reads (Day 2)	Total reads (Day 8)	Reads matching to sequence tags (%)	Wild-type reads (%)	Reads with NHEJ mutations (%)
RPL23 E-box exp 1	1578149	7408898	13.01 %	84.63 %	2.35 %
RPL23 E-box exp 2	3238187	22057987	9.26 %	89.23 %	1.51 %
HK2 E-box exp 1	6573471	9374627	9.40 %	89.98 %	0.62 %
HK2 E-box exp 2	5414108	24058374	7.66 %	91.76 %	0.58 %
PPAT E-box exp 1	4347450	26026333	23.47 %	68.12 %	8.41 %
PPAT E-box exp 2	1837186	31388024	26.75 %	63.65 %	9.61 %
MDN1 E-box 1	1344424	19241316	28.23 %	59.43 %	12.35 %
MDN1 E-box 2	901079	21667051	42.26 %	43.54 %	14.20 %
PAICS E-box exp 1	2872342	6110989	18.38 %	75.29 %	6.34 %
PAICS E-box exp 2	4835402	32720049	15.84 %	79.77 %	4.39 %
SHMT2 E-box exp 1	1436115	5331525	18.71 %	77.48 %	3.81 %
SHMT2 E-box exp 2	4338362	22578698	10.79 %	87.67 %	1.54 %
MYC negative control	1710381	21114866	10.68 %	83.02 %	6.30 %

Fitness experiments for phosphorylation sites

Experiment	Total reads (Day 2)	Total reads (Day 8)	Reads matching to sequence tags (%)	Wild-type reads (%)	Reads with NHEJ mutations (%)
CDK1 T161	6795702	9381740	1.19 %	89.59 %	9.22 %
CDK1 T14A/Y15F	8561775	8909263	4.00 %	72.07 %	23.93 %
CDK1-Y15F Prime editing	21504913	14381460	0.49 %	98.31 %	1.20 %
GRB2 160 HAP1	7925769	4217400	7.68 %	71.39 %	20.93 %
GRB2 209 HAP1	7157383	7556087	1.09 %	83.19 %	15.72 %
GRB2 160 KBM7	4397750	7307261	0.57 %	99.00 %	0.43 %
GRB2 209 KBM7	4080710	5555699	0.92 %	96.39 %	2.69 %

ChIP experiments for E-box targets

Experiment	Total reads (Input)	Total reads (MYC-IP)	Total reads (H3K27ac-IP)	Reads matching to sequence tags (%)	Wild-type reads (%)	Reads with NHEJ mutations (%)
HAP1 MDN1 ChIP	19998786	13992856	14905509	11.24 %	84.94 %	3.82 %
HAP1 RPL23 ChIP	13866486	14679295	15723855	7.87 %	90.31 %	1.82 %
HAP1 SHMT2 ChIP	16996331	29003166	28359009	8.50 %	90.32 %	1.18 %
HCT116 MDN1 ChIP	38392638	28321453	37787406	13.12 %	79.80 %	7.08 %
