# Supplementary material for: A competitive precision CRISPR method to identify the fitness effects of transcription factor binding sites
Source: Nat Biotechnol. 2022 Sep 26;41(2):197–203. doi: 10.1038/s41587-022-01444-6 (PMC9931575; doi:10.1038/s41587-022-01444-6)
Supplement: Supplementary file 2 — Reporting Summary [file 41587_2022_1444_MOESM2_ESM.pdf]

Corresponding author(s): Jussi Taipale

Last updated by author(s): Jul 13, 2022

Reporting Summary

Nature Research wishes to improve the reproducibility of the work that we publish. This form provides structure for consistency and transparency in reporting. For further information on Nature Research policies, see our Editorial Policies and the Editorial Policy Checklist.

Statistics

For all statistical analyses, confirm that the following items are present in the figure legend, table legend, main text, or Methods section.

n/a	Confirmed	
☐	☒	The exact sample size (*n*) for each experimental group/condition, given as a discrete number and unit of measurement
☐	☒	A statement on whether measurements were taken from distinct samples or whether the same sample was measured repeatedly
☐	☒	The statistical test(s) used AND whether they are one- or two-sided<br>*Only common tests should be described solely by name; describe more complex techniques in the Methods section.*
☐	☒	A description of all covariates tested
☐	☒	A description of any assumptions or corrections, such as tests of normality and adjustment for multiple comparisons
☐	☒	A full description of the statistical parameters including central tendency (e.g. means) or other basic estimates (e.g. regression coefficient) AND variation (e.g. standard deviation) or associated estimates of uncertainty (e.g. confidence intervals)
☐	☒	For null hypothesis testing, the test statistic (e.g. *F*, *t*, *r*) with confidence intervals, effect sizes, degrees of freedom and *P* value noted<br>*Give P values as exact values whenever suitable.*
☒	☐	For Bayesian analysis, information on the choice of priors and Markov chain Monte Carlo settings
☒	☐	For hierarchical and complex designs, identification of the appropriate level for tests and full reporting of outcomes
☐	☒	Estimates of effect sizes (e.g. Cohen's *d*, Pearson's *r*), indicating how they were calculated

Our web collection on statistics for biologists contains articles on many of the points above.

Software and code

Policy information about availability of computer code

Data collection	No specialized software was used for data acquisition, except for testing transfection efficiency using FACS with BD Influx System (USB) and BD FACS Sortware (version 1.2.0.142).
Data analysis	The following publicly available / previously published software were used in the data analysis as described and cited in the Methods: Guide sequences were designed using CRISPOR (version 4.99; http://crispor.tefor.net/). Sequencing reads were demultiplexed using bcl2fastq (version 2.20), and read number and quality was analyzed using FastQC (version 0.11.9). Preprocessing of the CGE data was performed using zgrep (version 1.10), grep (version 3.4), and agrep (version 3.0). Read counts for each unique sequence tag were counted using uniq -c (version 8.30). Sequence logos were generated using WebLogo (version 2.8.2; https://weblogo.berkeley.edu/logo.cgi). ChIP-seq reads were aligned to human genome (hg19) using bowtie2 (version 2.2.4), and peaks were called using MACS2 (version 2.1.1) against IgG or input with default narrow peak parameters. ATAC-seq fastq files were processed using an in-house pipeline comprising of following pieces of software: TrimGalore (version 0.4.3), BWA aligner (version 0.7.15), Picard (version 2.9.2) and broad-peak calling by MACS2 (version 2.1.1). RNA-seq reads were aligned to hg19 human genome using tophat2 (v2.0.13), and differentially expressed genes were analyzed using cuffdiff (v2.2.1). No custom code was used for the analyses.

For manuscripts utilizing custom algorithms or software that are central to the research but not yet described in published literature, software must be made available to editors and reviewers. We strongly encourage code deposition in a community repository (e.g. GitHub). See the Nature Research guidelines for submitting code & software for further information.

Data

Policy information about availability of data

All manuscripts must include a data availability statement. This statement should provide the following information, where applicable:

- Accession codes, unique identifiers, or web links for publicly available datasets
- A list of figures that have associated raw data
- A description of any restrictions on data availability

All next-generation sequencing data generated from the CGE assay as well as the HAP1 ATAC-seq data are available in European Nucleotide Archive (ENA) under accession number PRJEB52351. ChIP-seq data generated in this study is available under GEO accession GSE206080. Human genome sequence was used from Genome Reference Consortium Human Build 37 (GRCh37; hg19) under accession GCA_000001405. Previously published data sets for colon cancer cells were used as follows: RNA-seq from EGAD00001004098, ATAC-seq from GSE180158, and ChIP-nexus from EGAD00001004099.

Field-specific reporting

Please select the one below that is the best fit for your research. If you are not sure, read the appropriate sections before making your selection.

☒ Life sciences ☐ Behavioural & social sciences ☐ Ecological, evolutionary & environmental sciences

For a reference copy of the document with all sections, see nature.com/documents/nr-reporting-summary-flat.pdf

Life sciences study design

All studies must disclose on these points even when the disclosure is negative.

Sample size	CGE method generates a large number of internal replicates within each experiment. All internal replicates that met the inclusion criteria for the sequencing read count were included in the analyses, and thus sample size was not predetermined using statistical methods. The number of internal replicates used in each analysis is shown in Supplementary Table 3. The sample sizes used here are sufficient for making the conclusions about the effects of specific mutations, since independent experiments produced similar and statistically significant results.
Data exclusions	Cell lineages with negligible read counts in the day 2 samples of the fitness experiments or input samples of the ChIP experiments were excluded from the analysis as detailed in the Methods and Figure legends. The exclusion criteria were established before performing conclusion-related analyses.
Replication	The study design in the precision genome editing experiments creates a large number of internal replicate cultures within the single experiment, since the edited cell lineages can be analyzed separately due to their sequence tags, giving the assay high statistical power to detect phenotypic effects caused by targeted mutations. The results were reproducible in independent experiments as shown in Fig. 2d and Extended Data Fig. 7c. Moreover, internal replicate analysis confirmed the results as shown in Extended Data Fig. 7a,b. Replicate experiments were not performed for ChIP-CGE experiments, but similar results were obtained from two independent cell lines (see Fig. 2c and Extended Data Fig. 6).
Randomization	The CGE experiment results in a large number of cell lineages which can be analyzed independently based on their unique sequence tags. The key design feature of the CGE method is that the repair templates harboring two experimental variants (mutant and control) are transfected to the cells within one experiment, and can be analyzed as a pair, and thus randomization to experimental groups is not necessary. Therefore, all cell lineages that met the inclusion criteria were included in the analysis, and no randomization to different groups was done, except for the internal replicate analysis described below.<br><br>For internal replicate analysis shown in Fig. 2d and Extended Data Fig. 7a,b, individual cell lineages were randomized into two or four separate groups based on the mutations within their sequence tags. For randomizing the CDK1 T14A+Y15F lineages, the exact mutation of the first randomized nucleotide (see Supplementary Table 2) was used for separating the sequence tags into four groups having either A, C, T, or G in the first position. For E-box targets, the sequence tags for the HDR template libraries were generated by mutating each of the 10 nucleotides flanking the sequence of interest with the probability of 24%. The sequence tags with exactly two mutations were used in the analyses. For the purposes of the binning, the potentially mutated flanking positions were numbered from 1 to 10 in 5'-3' direction, and lineages were separated into two groups based on the first non-consensus nucleotide detected in each sequence tag (see Supplementary Table 1). The sequence tags with their first flanking mutation at odd position (1, 3, 5, 7, or 9) were grouped together, and the tags with their first flanking mutation at even position (2, 4, 6, 8) formed another group.
Blinding	The cell lineages were selected for analyses using predetermined inclusion criteria and analyzed using computer algorithms. Thus, it was not relevant for this study to blind the investigators for the study groups.

Reporting for specific materials, systems and methods

We require information from authors about some types of materials, experimental systems and methods used in many studies. Here, indicate whether each material, system or method listed is relevant to your study. If you are not sure if a list item applies to your research, read the appropriate section before selecting a response.

Materials & experimental systems

n/a	Involved in the study
☐	☒ Antibodies
☐	☒ Eukaryotic cell lines
☒	☐ Palaeontology and archaeology
☒	☐ Animals and other organisms
☒	☐ Human research participants
☒	☐ Clinical data
☒	☐ Dual use research of concern

Methods

n/a	Involved in the study
☐	☒ ChIP-seq
☐	☒ Flow cytometry
☒	☐ MRI-based neuroimaging

Antibodies

Antibodies used

Anti-MYC (#06-340, Millipore), anti-H3K27ac (#ab4729, Abcam), normal rabbit IgG (#sc-2027, SantaCruz); 5 ug of each antibody per immunoprecipitation reaction was used.

Validation

Anti-MYC (#06-340, Millipore) is a rabbit polyclonal antibody validated for ChIP by the manufacturer. There are >50 previous publications using this antibody, as shown in the manufacturer's website https://www.merckmillipore.com/FI/en/product/Anti-Myc-Antibody,MM_NF-06-340?ReferrerURL=https%3A%2F%2Fwww.google.com%2F&bd=1#documentation. The antibody is raised against bacterially expressed fusion-protein corresponding to the full-length human Myc.

Anti-H3K27ac (#ab4729, Abcam) is a rabbit polyclonal antibody to histone H3 (acetyl K27) raised against a synthetic peptide corresponding to Human Histone H3 aa 1-100 (acetyl K27) conjugated to keyhole limpet haemocyanin. The antibody is validated as ChIP grade by the manufacturer and has over 1,400 citations on the manufacturer's website https://www.abcam.com/histone-h3-acetyl-k27-antibody-chip-grade-ab4729.html.

Normal IgGs from Santa Cruz are commonly used controls in ChIP experiments. Normal rabbit IgG is an unconjugated, affinity purified isotype control immunoglobulin from rabbit, and it is s recommended by the manufacturerfor to be used as a isotype control immunoglobulin in place of a target-specific primary antibody of the same isotype (rabbit IgG). The antibody has been used in several publication, as listed for example here: https://datasheets.scbt.com/sc-2027.pdf

Eukaryotic cell lines

Policy information about cell lines

Cell line source(s)

HAP1 and KBM-7 cell lines were obtained from Horizon Discovery (#C631 and #C628, respectively). HCT116 cell line (#CCL-247) was obtained from ATCC

Authentication

Cell lines were directly obtained from a trusted vendor (Horizon Discovery, ATCC), and only low-passage cells were used in the experiments. Cell lines were not authenticated.

Mycoplasma contamination

All cell lines used in this study were tested negative for mycoplasma contamination upon purchase and were routinely monitored thereafter.

Commonly misidentified lines
(See ICLAC register)

No commonly misidentified cell lines were used in this study.

ChIP-seq

Data deposition

☒ Confirm that both raw and final processed data have been deposited in a public database such as GEO.

☒ Confirm that you have deposited or provided access to graph files (e.g. BED files) for the called peaks.

Data access links
May remain private before publication.

ChIP-seq data generated in this study is available under GEO accession GSE206080, and both raw and processed files are available.

Files in database submission

HAP1_H3K27ac_S18_R1_001.fastq.gz
HAP1_rIgG_S16_R1_001.fastq.gz
HAP1_MYC_S27_R1_001.fastq.gz
HAP1_Input_S29_R1_001.fastq.gz
HAP1_H3K27ac_vs_rIgG_peaks.narrowPeak
HAP1_MYC_vs_Input_peaks.narrowPeak
HAP1_H3K27ac.bdg
HAP1_rIgG.bdg
HAP1_MYC.bdg
HAP1_Input.bdg

Genome browser session
(e.g. UCSC)

No longer applicable

Methodology

Replicates	ChIP-seq data was used for confirming MYC and H3K27ac signals in HAP1 cells but not for any quantitative analyses. For that reason, no replicates were used.
Sequencing depth	Mapped reads for H3K27ac; total = 42441091; unique = 31981022<br>Mapped reads for IgG control used for H3K27ac; total = 35684976; unique = 23541240<br>Mapped reads for MYC; total = 38975262; unique =14272022<br>Mapped reads for input control used for MYC; total = 27417288; unique = 22898905
Antibodies	Anti-MYC (#06-340, Millipore), anti-H3K27ac (#ab4729, Abcam), normal rabbit IgG (#sc-2027, SantaCruz).
Peak calling parameters	Peak calling was performed using MACS2 with default parameters.
Data quality	Correct TF motifs identified within the peaks.
Software	Bowtie2 (Langmead, & Salzberg, Nat Methods 9, 357-359, 2012)<br>MACS2 (Zhang et al., Genome Biol. 9, pp. R137, 2008)

Flow Cytometry

Plots

Confirm that:

☒ The axis labels state the marker and fluorochrome used (e.g. CD4-FITC).

☒ The axis scales are clearly visible. Include numbers along axes only for bottom left plot of group (a 'group' is an analysis of identical markers).

☒ All plots are contour plots with outliers or pseudocolor plots.

☒ A numerical value for number of cells or percentage (with statistics) is provided.

Methodology

Sample preparation	Human HAP1 cells were transfected with ribonucleoprotein (RNP) complex, for which sgRNA molecules were generated by annealing equimolar ratios of target-specific crRNAs and ATTO550-tracrRNA (Integrated DNA Technologies). RNP complexes were constituted from S.p. HiFi Cas9-protein (Integrated DNA Technologies; 1000 ng / 200,000 cells) and target-specific sgRNA (250 ng / 200,000 cells) and transfected to cells using CRISPRMAX (Life Technologies) as per manufacturer's recommendation along with HDR template (1:1 mixture of the original and mutant HDR templates) with final concentration of 3 nM. Cells were harvested for flow cytometry analysis 24 h after transfection by trypsination, washed once with cold PBS, and resuspended in cold PBS. Just before sorting, cells were passed through a 35 nm strainer and mixed with SYTOX Blue dead cell stain (Invitrogen) according to manufacturer's instructions. The flow cytometry analysis was performed at the HiLife Flow Cytometry Unit, University of Helsinki, Finland, using BD Influx System (USB) and BD FACS Sortware (version 1.2.0.142).
Instrument	BD Influx System (USB), model number #X646500S7001
Software	BD FACS™ Sortware, version 1.2.0.142
Cell population abundance	Out of 84,191 transfected cells analyzed, 79.26% were singlets based on SSC/FSC, out of which 70.99% were live based on the Sytox dead cell indicator (Invitrogen). Gate for ATTO550 was set so that all non-transfected cells were negative. Out of 70,995 live cells analyzed from the transfected sample, 40.53% were positive for ATTO550.
Gating strategy	Manual gating was performed using non-transfected control cells, and similar gates were applied for transfected samples to analyze transfection efficiency. The gating strategy is exemplified in Extended Data Fig. 9 (top panels). 1. FSC/SSC: Cells were gated on the main population, excluding clear outliers such as cell debris. 2. FSC/Trigger pulse width: Cells were gated on the main population that represent single cells, excluding the outliers with larger trigger pulse width representing potential duplets. 3. FSC/405 nm (ex. 405nm, em. 460/50nm for SYTOX Blue dead cell stain): Viability was checked using the SYTOX stain and SYTOX-negative cells were gated to exclude the dead cells with higher fluorescence values. 4. Fluorescence was monitored on two channels (ex. 488 nm, em. 530/40 nm as an extra negative control) and ex. 561 nm, em. 585/29 nm for ATTO550. Gate was set using the non-transfected HAP1 cells so that all cells remained negative for ATTO550. Same gate was maintained to analyze transfected cells to measure the proportion of ATTO550-positive cells.

☒ Tick this box to confirm that a figure exemplifying the gating strategy is provided in the Supplementary Information.
